# Disulfidptosis: Mechanisms, evidence boundaries, and translational opportunities

**DOI:** 10.1016/j.redox.2026.104306

**Published:** 2026-07-16

**Authors:** Rongqing Li, Jiahui Wang, Wei Li, Li Qian

**Affiliations:** a Key Laboratory of the Jiangsu Higher Education Institutions for Nucleic Acid & Cell Fate, Regulation (Yangzhou University), School of Basic Medical Sciences & School of Public Health, Faculty of Medicine, Yangzhou University, Yangzhou, 225009, PR China; b Department of Center Laboratory, Kunshan Hospital of Chinese Medicine, Affiliated Hospital of Yangzhou University, Kunshan, Jiangsu, 215300, PR China

**Keywords:** Disulfidptosis, Disulfide stress, SLC7A11, Actin cytoskeleton, Metabolic vulnerability

## Abstract

Disulfidptosis has rapidly emerged as a regulated cell death mechanism linked to cystine stress, aberrant disulfide accumulation, and collapse of the actin cytoskeleton, particularly in contexts shaped by high SLC7A11 activity and impaired NADPH-dependent reducing capacity. However, the literature has expanded faster than the mechanistic standards used to classify this process, and studies invoking disulfidptosis now range from direct experimental demonstrations to purely association-based bioinformatic analyses. This heterogeneity creates a growing risk of overassignment of the term and blurs the boundary between bona fide disulfidptosis and related redox or metabolic stress phenotypes. In this review, we prioritize studies that provide direct mechanistic support for disulfidptosis and propose a practical evidence-tier framework for mechanistic assignment. Rather than treating all “disulfidptosis-related” reports as equivalent, we distinguish high-confidence evidence from inferential or hypothesis-generating observations and discuss the interpretive limitations of lower-tier claims. We synthesize current knowledge on the biochemical basis, cellular prerequisites, morphological and molecular hallmarks, experimental readouts, and disease contexts of disulfidptosis. By emphasizing rigorous evidence interpretation and integrating multi-omics prediction, mechanistic crosstalk, and clinically tractable therapeutic strategies, this review outlines the current conceptual boundaries of disulfidptosis and offers a useful reference for future mechanistic research and translational development.

## Highlights

• Disulfidptosis is driven by cystine stress and actin cytoskeletal collapse.
• We clarify exact mechanistic boundaries to prevent concept overassignment.
• A three-tier evidence framework is proposed for rigorous mechanistic assignment.
• Redox-targeted translational therapies are evaluated using this framework.

## Graphical abstract

**Figure:**
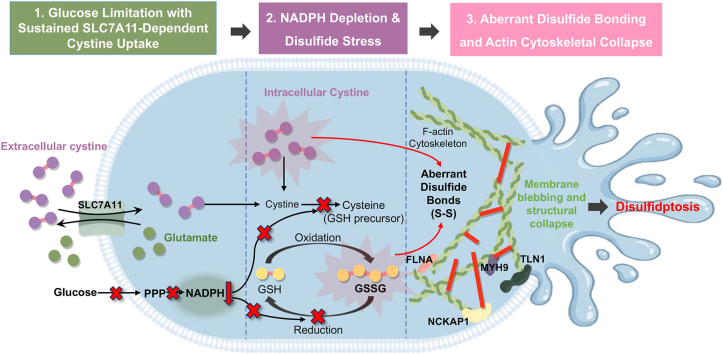

## 1 Introduction

Disulfidptosis has emerged as a distinct form of regulated cell death linked to metabolic stress and redox imbalance, attracting increasing attention since its initial description [[1], [2], [3], [4], [5]]. Unlike conventional cell death paradigms defined primarily by morphology or canonical signaling modules [[6], [7], [8], [9], [10], [11]], disulfidptosis is notable for its dependence on cystine overload under conditions of limited reducing capacity, leading to abnormal disulfide stress and the collapse of the actin cytoskeleton. This mechanistic emphasis has made the concept particularly relevant to tumors with high solute carrier family 7 member 11 (SLC7A11) expression and altered glucose metabolism, while also raising broader questions regarding whether similar vulnerabilities may operate in other pathological settings [[12], [13], [14]]. As interest in the field expands, the literature has become increasingly heterogeneous in terminology, mechanistic rigor, and disease extrapolation. In many instances, findings suggestive of oxidative stress, altered sulfur metabolism, or treatment sensitivity have been interpreted as evidence of disulfidptosis without demonstrating the defining molecular sequence of events. These inconsistencies necessitate a closer examination of the field with greater conceptual precision. The critical issue now is not simply whether disulfidptosis can be invoked in an expanding range of biological contexts, but whether the term is being applied in a manner that remains mechanistically grounded. A clearer framework is required because the field sits at the intersection of metabolism, redox homeostasis, cytoskeletal biology, and disease pathology, yet the boundaries between direct evidence and broader association are not always rigorously maintained. The strongest current evidence supports a model in which excessive cystine uptake, when coupled to impaired reductive buffering, promotes aberrant disulfide accumulation and the destabilization of actin cytoskeletal structures, ultimately driving cell death [1,15,16]. Concurrently, numerous questions remain unresolved, including the upstream determinants of susceptibility, the hierarchy of participating molecules, the extent of crosstalk with other stress response pathways, and the degree to which observations made in tumor systems can be generalized to non-neoplastic diseases.

In this Review, we therefore adopt a focused, evidence-based approach centered on mechanistic assignment. We prioritize studies that directly inform the defining biochemical, cellular, and phenotypic features of bona fide disulfidptosis, and we use a practical evidence-tier framework to distinguish high-confidence mechanistic support from more inferential forms of evidence. Lower-confidence reports are discussed more selectively, primarily to illustrate interpretive boundaries, unresolved questions, and areas where experimental validation is still needed. By helping to clarify how bona fide disulfidptosis is presently understood, we aim to support greater conceptual consistency in the field and offer a useful reference point for future mechanistic and translational studies.

## 2 Literature curation and evidence grading approach

To improve transparency and interpretive consistency, the literature discussed herein was curated utilizing a structured approach. We searched PubMed, Web of Science, and Google Scholar for studies published up to March 2026 using combinations of terms including disulfidptosis, SLC7A11, cystine uptake, disulfide stress, glucose starvation, glucose transporter (GLUT) inhibition, actin cytoskeleton, and regulated cell death. Studies were subsequently classified interpretively into three evidence tiers based on the extent of mechanistic support. This classification considered the metabolic context, dependence on cystine transport or SLC7A11 activity, evidence of disulfide stress, documentation of actin cytoskeletal disruption, response to mechanistically relevant rescue interventions, and the exclusion of alternative death programs. Because the field remains relatively nascent and experimental approaches vary substantially across models, this grading system is intended to serve as a practical interpretive framework rather than a formal evidence scoring instrument.

## 3 What qualifies as disulfidptosis?

As interest in disulfidptosis has proliferated, the term has begun to appear in experimental settings that differ substantially in mechanistic rigor. This is perhaps unsurprising in a field that is still taking shape, but it creates a distinct problem: if the label is applied too broadly, it becomes difficult to ascertain whether a study is documenting disulfidptosis itself or a more generalized form of redox-related cell injury. For this reason, we consider it necessary to state explicitly what type of evidence supports a mechanistic assignment of disulfidptosis and what type of evidence should still be regarded as provisional.

At present, disulfidptosis is best treated as a mechanism-based designation rather than a descriptive label. In practical terms, this means that assignment should rest on converging evidence derived from the perturbation context, the pattern of pathway dependence, the nature of the structural damage, and the response to interventions that are mechanistically relevant. Studies are most persuasive when cell death occurs under conditions expected to compromise reductive buffering in cells with high cystine uptake, when the phenotype depends on cystine transport or SLC7A11 activity rather than expression alone, when aberrant disulfide stress can be definitively demonstrated, and when actin cytoskeletal damage is directly documented. The evidence becomes even stronger when the phenotype can be prevented or attenuated by reducing agents, by limiting cystine uptake, or by restoring redox balance, provided that alternative death programs are reasonably excluded [17,18].

These considerations are not intended to function as a rigid checklist. In many experimental systems, particularly in vivo models, not every feature can be examined with the same degree of precision. Nevertheless, the mechanistic case is clearly stronger when several defining elements are demonstrated concurrently than when the conclusion is inferred from a single upstream marker or a broadly stress-related phenotype. Conversely, caution is warranted when the evidence relies solely on susceptibility factors or correlative observations.

Certain findings, although suggestive, should not be considered sufficient in isolation. High SLC7A11 expression does not independently establish that disulfidptosis has occurred [11,[18], [19], [20]]. Furthermore, glucose starvation-induced cell death is insufficient if disulfide accumulation and actin cytoskeletal alterations are not directly examined [[21], [22], [23], [24]]. The same caution applies to transcriptomic signatures, correlative biomarker studies, or pathway inferences drawn from gene expression datasets. These approaches may indicate a setting wherein disulfidptosis is plausible, but they do not demonstrate that it is the operative death mechanism. Likewise, rescue by broad antioxidants alone is difficult to interpret, as such interventions may blunt oxidative injury in a nonspecific manner without confirming that disulfide-driven cytoskeletal collapse has been reversed.

For the purposes of this review, we therefore categorize the literature into three evidence Tiers. Tier 1 encompasses studies providing direct mechanistic support for disulfidptosis, including an appropriate metabolic context, dependence on cystine uptake or SLC7A11 activity, evidence of disulfide stress, documentation of actin cytoskeletal disruption, meaningful rescue by relevant interventions, and reasonable exclusion of competing death programs. Tier 2 encompasses studies that support components of this model but leave critical mechanistic steps unresolved. This category includes research demonstrating selective vulnerability linked to cystine metabolism or redox imbalance, but lacking direct demonstration of the structural execution phase. Tier 3 encompasses association-based evidence, including transcriptomic analyses, biomarker correlations, and descriptive observations that are compatible with disulfidptosis but fail to establish it mechanistically. We adopt this tiered framework not to impose rigid boundaries on an emerging field, but to reduce conceptual ambiguity and to distinguish validated conclusions from more provisional or associative interpretations (Table 1).

**Table 1:** Practical evidence tiers for mechanistic assignment of disulfidptosis.

Evidence tier	What the evidence usually includes	Interpretive implication	Common limitations
**Tier 1**	Cell death arising in a metabolically relevant context; dependence on cystine uptake or SLC7A11 activity; direct or closely linked evidence of disulfide stress; documentation of actin cytoskeletal disruption; meaningful rescue by mechanistically relevant interventions (e.g., reducing agents); reasonable exclusion of alternative death programs.	Relatively strong mechanistic support that disulfidptosis is the operative mode of cell death in the tested setting.	Not every study can examine all steps with equal depth; some evidence may still rely on surrogate readouts; exclusion of alternative death programs is sometimes incomplete.
**Tier 2**	Partial support for the model, such as selective vulnerability associated with cystine metabolism, glucose limitation, redox imbalance, or SLC7A11 dependence, but without direct demonstration of disulfide accumulation and/or cytoskeletal collapse.	The findings are consistent with disulfidptosis and indicate that the pathway or related metabolic vulnerabilities are involved.	Key mechanistic links remain unresolved; the terminal structural execution process is not directly established; overlap with other stress-associated death programs cannot be excluded.
**Tier 3**	Association-based evidence, including transcriptomic signatures, biomarker correlations, expression analyses, descriptive phenotypes, or pathway inferences without direct mechanistic testing.	Suggests that disulfidptosis-related networks may be biologically or clinically relevant in a given context.	Does not establish disulfidptosis as the death mechanism; highly vulnerable to overinterpretation; often unable to distinguish disulfidptosis from broader redox stress.

Table 1:The evidence tiers summarized here are intended as a practical interpretive framework for this review rather than as a formal grading system. Assignment to Tier 1 reflects the overall strength of mechanistic convergence rather than identical experimental execution across all models. High SLC7A11 expression, simple antioxidant sensitivity, or transcriptomic associations alone should not be considered sufficient evidence for definitive disulfidptosis.

Following the three-tier evidence framework, we suggest that a definitive mechanistic assignment of disulfidptosis should require convergent evidence across several experimental dimensions. At a minimum, investigators should demonstrate that cell death is linked to cystine loading, for example by showing preferential vulnerability in SLC7A11-high or cystine-importing cells and rescue through cystine withdrawal, SLC7A11 knockdown, or inhibition of cystine uptake. This should be accompanied by evidence that cellular reducing capacity is compromised under the death-inducing condition, such as NADPH depletion, GSH/GSSG imbalance, or accumulation of intracellular disulfides. Because disulfidptosis is mechanistically defined by disulfide stress-induced cytoskeletal damage, direct assessment of the actin cytoskeleton should also be included, ideally showing actin network collapse together with increased disulfide bonding, aggregation, or aberrant modification of cytoskeletal proteins. Rescue experiments should be mechanism-relevant, such as limiting cystine influx or restoring reducing equivalents, rather than relying solely on nonspecific antioxidant protection. Finally, major competing regulated cell death programs, including apoptosis, necroptosis, ferroptosis, and other context-relevant modalities, should be excluded using orthogonal pharmacological, genetic, and biochemical approaches. Accordingly, high SLC7A11 expression, transcriptomic signatures, glucose starvation-induced death, or rescue by broad antioxidants alone should be considered supportive but insufficient to establish disulfidptosis.

## 4 Disulfidptosis and other regulated cell death modalities

### 4.1 Apoptosis

The central execution mechanism of apoptosis relies on initiator and effector caspases, which lead to membrane blebbing, nuclear condensation and DNA fragmentation [25]. In cells with high SLC7A11 expression that experience glucose starvation or acute disruption of glucose flux, disulfidptosis proceeds even in the presence of a pan caspase inhibitor [1]. The predominant phenotype of this process is actin disulfide crosslinking and subsequent cytoskeletal collapse instead of caspase-dependent nuclear fragmentation [1]. These findings demonstrate clear mechanistic distinction between disulfidptosis and apoptosis through direct perturbation and rescue experiments. No published studies have shown that canonical apoptotic machinery directly regulates disulfidptosis beyond generic stress-associated responses, so any claimed connection is supported only by indirect correlation or computational inference without direct causal testing.

### 4.2 Necroptosis

Necroptosis is driven by a core machinery involving Receptor-interacting serine/threonine-protein kinase 3 (RIPK3)-mediated phosphorylation of MLKL, which then oligomerizes to permeabilize the plasma membrane [26]. The founding study on disulfidptosis reported that RIPK1 inhibition failed to rescue this cell death modality, and no involvement of Mixed lineage kinase domain-like protein (MLKL) was observed. Additionally, classical readouts of necroptosis have not been implicated in disulfidptosis [1]. Collectively, current experimental evidence suggests key distinctions between the two processes, with limited validated positive regulatory interplay identified to date. Accordingly, any potential association between them remains largely supported only by indirect evidence.

### 4.3 Pyroptosis

Pyroptosis is characterized by inflammatory caspases cleaving gasdermin D to form membrane pores, thereby promoting interleukin release [27]. No direct evidence has been found that gasdermin D is activated during disulfidptosis, nor have specific pyroptosis readouts been shown to be part of the execution process of disulfidptosis [1]. As such, the regulatory relationship between pyroptosis and disulfidptosis still warrants further clarification. Existing evidence for their potential linkage is largely limited to indirect observations and computational predictions.

### 4.4 Ferroptosis

Ferroptosis is an iron-dependent cell death modality, triggered by uncontrolled phospholipid peroxidation that is restrained by glutathione peroxidase 4 (GPX4) and lipid peroxide scavengers [11,28]. Disulfidptosis can proceed normally in the presence of ferroptosis inhibitors and iron chelators, and it lacks the biochemical signature of lethal lipid peroxidation during its execution window. This observation provides direct experimental evidence for the distinction between the two [1].

At the same time, the two pathways are closely linked through a shared redox-metabolic network centered on SLC7A11, cystine/cysteine metabolism, glutathione, and NADPH [1]. Under nutrient-replete conditions, SLC7A11-mediated cystine uptake supports cysteine availability, glutathione synthesis, and GPX4-dependent detoxification of lipid peroxides, thereby suppressing ferroptosis [11,28]. Under glucose-limited conditions, however, elevated cystine uptake can become maladaptive because intracellular cystine reduction imposes a substantial NADPH demand. When NADPH regeneration is insufficient, disulfide stress accumulates and promotes actin disulfide crosslinking, ultimately driving disulfidptosis [1,11,28]. These observations indicate that ferroptosis and disulfidptosis are mechanistically distinct at the level of execution, yet closely connected through a shared upstream redox-metabolic framework centered on SLC7A11, GSH, NADPH, and cystine metabolism.

### 4.5 Cuproptosis

Cuproptosis is triggered by copper binding to lipoylated tricarboxylic acid cycle proteins, which promotes their aggregation and loss of function. This process is dependent on Ferredoxin 1 (FDX1) and protein lipoylation [29,30]. Unlike disulfidptosis, which is driven by cystine overload, impaired reducing capacity and actin cytoskeletal disulfide damage, cuproptosis is primarily executed through mitochondrial protein toxicity [29]. The two pathways may nevertheless intersect through the SLC7A11/GSH axis, as SLC7A11-derived cysteine supports GSH synthesis and GSH can buffer intracellular copper via thiol-dependent binding [31]. Therefore, cuproptosis should be considered a potential competing death modality in SLC7A11-high cells, but its assignment requires evidence of copper-dependent mitochondrial proteotoxicity rather than disulfide-mediated cytoskeletal collapse.

### 4.6 Autophagy-dependent cell death

Lethal outcomes of autophagy-dependent cell death require the functions of core Autophagy-related genes (ATGs), and this death modality can be diagnosed when genetic ablation of autophagy blocks cell death [32]. Disulfidptosis has not been shown to require ATGs, and its proximal effector is actin disulfide crosslinking coupled with the loss of lamellipodial dynamics. Reviews have proposed that autophagy may secondarily regulate redox and NADPH homeostasis, which could influence susceptibility to disulfidptosis, but these suggestions have not yet been supported by causal tests [15,16,33]. Current evidence regarding their functional linkage remains largely indirect, and direct experimental data verifying a definitive dependence is still lacking.

### 4.7 Parthanatos

Parthanatos is a caspase-independent form of regulated cell death triggered by excessive PARP1 activation under oxidative stress or severe DNA damage, leading to poly(ADP-ribose) accumulation, Nicotinamide adenine dinucleotide (NAD^+^) depletion, and apoptosis-inducing factor (AIF) translocation from mitochondria to the nucleus [[34], [35], [36]]. In the nucleus, AIF cooperates with associated nucleases to drive large-scale DNA fragmentation, thereby executing parthanatos [[34], [35], [36]]. No published studies have demonstrated that disulfidptosis requires PARP1 activation or AIF translocation, and the actin disulfide model does not involve a nuclear nuclease step. Accordingly, potential associations between parthanatos and disulfidptosis are currently supported mainly by indirect evidence, while solid causal evidence remains insufficient.

### 4.8 Integrative view of disulfidptosis and other death modalities

Taken together, disulfidptosis should be viewed as a distinct redox-metabolic cell death modality that intersects with, but is not equivalent to, other regulated cell death programs. Its closest mechanistic overlap is with ferroptosis through the SLC7A11-cystine/cysteine-GSH-NADPH-GPX4 network, whereas its potential connection with cuproptosis is more indirect and may involve copper–thiol interactions, GSH-dependent metal buffering and mitochondrial redox stress. Beyond these metabolic intersections, disulfidptosis may coexist with or secondarily influence apoptosis, necroptosis, pyroptosis and autophagy-related responses under severe redox or energetic stress. However, these death modalities are defined by distinct execution mechanisms, including caspase activation, MLKL-dependent membrane rupture, inflammasome-mediated pore formation, or lysosomal/autophagic dysfunction, none of which constitutes the defining event of disulfidptosis.

**Fig. 1:**
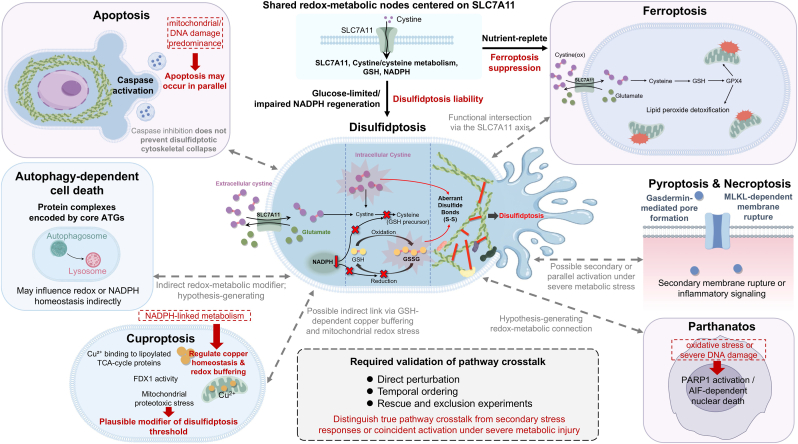
Conceptual map of disulfidptosis and its relationship to other regulated cell death modalities.

Therefore, pathway crosstalk should not be mistaken for pathway identity. Shared involvement of SLC7A11, GSH, NADPH, oxidative stress or metabolic vulnerability may indicate overlapping stress responses, but it is insufficient to assign a specific death modality. Mechanistic assignment of disulfidptosis requires convergent evidence for cystine-loading susceptibility, impaired reducing capacity, intracellular disulfide accumulation, actin cytoskeletal disulfide damage, mechanism-relevant rescue and exclusion of major competing death programs, particularly ferroptosis, cuproptosis, apoptosis and necroptosis (Fig. 1).

The central panel summarizes the canonical disulfidptosis pathway, in which high SLC7A11-dependent cystine uptake under glucose-limited conditions drives NADPH depletion, promotes actin disulfide stress, and ultimately leads to F-actin cytoskeletal collapse. The upper panel highlights potentially shared metabolic and redox nodes across regulated cell death pathways, with the SLC7A11 axis representing the closest functional intersection. The surrounding comparison panels outline the defining features of apoptosis, ferroptosis, autophagy-dependent cell death, cuproptosis, pyroptosis, necroptosis, and parthanatos, and indicate where current evidence supports distinction, partial overlap, or putative context-dependent connection with disulfidptosis. The lower validation panel summarizes the experimental framework required to distinguish direct mechanistic crosstalk from indirect association, including perturbation, temporal analysis, and rescue and exclusion experiments.

## 5 Regulatory landscape of disulfidptosis

### 5.1 Core regulatory logic of disulfidptosis

Current evidence indicates that its regulatory architecture converges on three interconnected layers: (i) control of cystine transport, especially through SLC7A11, the major cystine/glutamate antiporter [18]; (ii) maintenance or exhaustion of nicotinamide adenine dinucleotide phosphate (NADPH)-dependent reducing power, largely sustained by glucose metabolism and the pentose phosphate pathway (PPP) [1,18,[37], [38], [39], [40], [41]]; and (iii) execution-phase modulation of actin cytoskeletal integrity under disulfide overload, which determines whether metabolic stress is ultimately converted into structural collapse [1,42,43]. Rather than being driven by a single gene, disulfidptosis appears to emerge from the dynamic interaction between these layers [1].

Although current evidence supports actin cytoskeletal disulfide damage as a central execution-related event in disulfidptosis, important mechanistic questions remain unresolved [3]. In particular, it is unclear whether all actin-associated disulfide-crosslinking events contribute comparably to cytoskeletal collapse, or whether a smaller subset of structurally strategic “sentry” proteins plays a dominant causal role in driving the lethal phenotype. Current studies have identified multiple candidate substrates, including actin and several actin-associated regulators, but detection of disulfide crosslinking alone does not establish causal hierarchy [15]. Future studies that combine temporal redox proteomic profiling with functional perturbation approaches may help clarify whether specific redox-sensitive actin-associated proteins occupy a causal position in cytoskeletal collapse. For example, redox-resistant mutants or targeted rescue strategies could be used to examine whether selective modulation of candidate cysteine residues attenuates F-actin disruption and subsequent cell death.

**Fig. 2:**
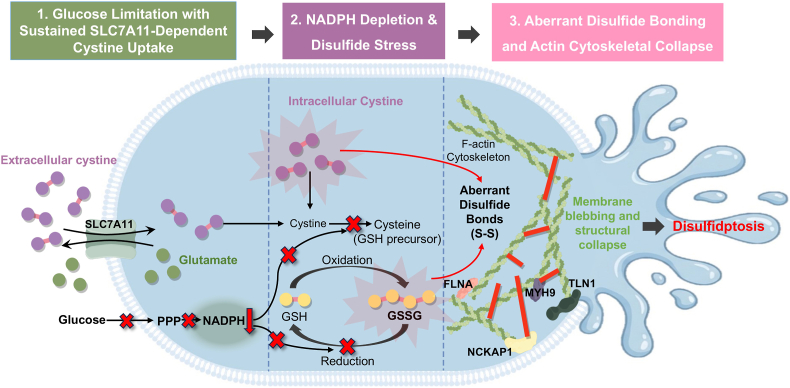
Core mechanistic cascade of disulfidptosis under sustained cystine uptake and impaired reducing capacity.

The currently proposed core biochemical and structural cascade of disulfidptosis is summarized in Fig. 2. Representative actin-associated proteins depicted in the figure, such as non-muscle myosin heavy chain IIA (MYH9), NCK-associated protein 1 (NCKAP1), and talin 1 (TLN1), are included as commonly reported cytoskeletal proteins associated with disulfide stress-induced actin remodeling.

Within this framework, SLC7A11-centered regulation remains the most firmly established framework. Cells with high SLC7A11 expression are particularly vulnerable to glucose starvation or GLUT inhibition because sustained cystine uptake imposes a heavy reducing burden that cannot be resolved when NADPH production collapses [18,44,45]. However, susceptibility is not determined by SLC7A11 alone. Multiple transcriptional, post-transcriptional, epigenetic, and metabolic regulators can raise or lower the threshold for lethal disulfide accumulation by altering either cystine influx or the capacity to regenerate reducing equivalents. This broader view is essential for understanding why disulfidptosis exhibits substantial context dependence across tumor types, nutrient states, and disease settings. This context dependence is also reflected in the divergent consequences of SLC7A11 activity across distinct metabolic and cell death settings [[46], [47], [48], [49]].

Disulfidptosis is a metabolic-cytoskeletal form of regulated cell death triggered when continued SLC7A11-dependent cystine uptake is coupled to insufficient reducing power, most prominently during glucose starvation or acute restriction of glucose utilization. Under these conditions, reduced pentose phosphate pathway flux limits NADPH regeneration, thereby impairing cystine reduction and promoting intracellular disulfide stress. Excess disulfide burden drives aberrant inter- and intramolecular disulfide bonding within the actin cytoskeleton and associated structural proteins. The cytoskeletal components depicted in this scheme include F-actin together with actin-associated proteins such as FLNA, MYH9, TLN1, and NCKAP1. These structural changes culminate in F-actin crosslinking, cytoskeletal contraction, membrane detachment, and lethal cellular collapse.

### 5.2 Transcriptional programs controlling disulfidptotic susceptibility

At the transcriptional level, regulators of disulfidptosis can be functionally grouped according to the three core determinants defined above: cystine transport, reductive buffering capacity, and cytoskeletal susceptibility to disulfide stress. This framework helps distinguish transcriptional events that increase the upstream cystine burden from those that preserve reducing power or alter the efficiency of downstream structural collapse. Rather than functioning as isolated events, these transcriptional programs collectively determine whether persistent cystine uptake can be buffered metabolically or instead converted into lethal disulfide stress.

#### 5.2.1 SLC7A11

Among transcriptional determinants of disulfidptosis, SLC7A11 remains the most direct and best-established regulatory node. Transcriptional activation of SLC7A11 generally increases susceptibility to disulfidptosis under glucose-limiting conditions by sustaining cystine import beyond the capacity of the NADPH-generating network, whereas repression of SLC7A11 lowers this burden and confers protection.

A representative example has been described in clear cell renal cell carcinoma (ccRCC), where BRCA1-associated protein 1 (BAP1) acts as a negative regulator of disulfidptosis [50]. Previous studies have already established BAP1 as a regulator of other cell death programs, including apoptosis and ferroptosis [[51], [52], [53], [54]], supporting a broader role for this tumor suppressor in cell fate control. BAP1 suppresses SLC7A11 transcription by reducing H2A ubiquitination at the SLC7A11 promoter in a deubiquitinase-dependent manner. This model was further supported by the finding that enforced SLC7A11 expression weakened BAP1-mediated protection, whereas SLC7A11 deletion or erastin treatment abolished the vulnerability of BAP1-low cells, consistent with reduced cystine influx as the key upstream determinant. Together, these data support a BAP1/SLC7A11/NADPH axis as an important transcriptional program regulating disulfidptotic sensitivity in ccRCC. This example illustrates how transcriptional repression of SLC7A11 can reduce the initial substrate burden that drives the entire death cascade.

A second well-defined example comes from prostate cancer, in which a super-enhancer/FOXA1/SLC7A11 axis establishes another direct transcriptional route to disulfidptosis resistance [55]. Forkhead box A1 (FOXA1) is a lineage-defining pioneer transcription factor that reshapes chromatin accessibility and coordinates hormone-responsive transcriptional programs, particularly in prostate cancer [[56], [57], [58]]. A super-enhancer located at chromosome 14 drives FOXA1 expression, and FOXA1 directly binds the SLC7A11 promoter to activate its transcription. High SLC7A11 expression maintains cystine uptake and glutathione homeostasis, whereas glucose deprivation or BAY-876-mediated GLUT1 (glucose transporter 1) inhibition disrupts NADPH generation, leading to disulfide stress, actin cytoskeletal collapse, and disulfidptosis [55]. Genetic ablation of the super-enhancer, FOXA1 inhibition, or restriction of cystine uptake reverses this phenotype independently of apoptosis or ferroptosis. This study underscores how enhancer architecture can define disulfidptotic vulnerability by tuning the SLC7A11 axis [55].

In bone biology, disulfidptosis-like mechanisms have also been mechanistically defined. Nuclear factor of activated T cells 1 (NFATc1) is a master transcription factor for osteoclast differentiation and activation [[59], [60], [61]], whereas TrxR1 is a key thioredoxin system oxidoreductase that maintains intracellular thiol redox homeostasis by reducing oxidized thioredoxin [[62], [63], [64]]. In pre-osteoclasts, NFATc1 transcriptionally activates SLC7A11, enhancing cystine uptake, while TrxR1 inhibition blocks disulfide reduction, causing cystine accumulation, disulfide stress, F-actin contraction, and disulfidptosis [65]. This phenotype can be rescued by SLC7A11 inhibition or disulfide-reducing agents, and competing death pathways were excluded.

Other transcriptional candidates are supported by more intermediate-level evidence. In breast cancer, circular RNA 0022382 (circ_0022382), induced by eukaryotic translation initiation factor 4A3 (EIF4A3), sponges let-7a-5p, activates the PI3K/AKT/mTOR pathway (comprising phosphoinositide 3-kinase, AKT serine/threonine kinase, and mechanistic target of rapamycin kinase), and upregulates SLC7A11, enhancing cystine uptake and thereby increasing the burden on cellular reducing capacity. Its knockdown lowers NADPH levels and increases sensitivity to glucose starvation-induced death; however, direct evidence for canonical disulfidptotic hallmarks, such as actin-associated disulfide accumulation and F-actin collapse, remains limited [66]. Thus, circ_0022382 is better regarded as a probable modulator of the SLC7A11 arm rather than a fully validated core regulator.

Together, these findings indicate that direct transcriptional control of SLC7A11 primarily governs the substrate-loading phase of disulfidptosis. By determining the baseline intensity of cystine influx, this layer sets the metabolic burden that downstream glucose and NADPH buffering systems must counterbalance. For this reason, direct SLC7A11 regulation currently represents the most proximal and best-validated transcriptional entry point into the disulfidptotic network.

#### 5.2.2 PPP/NADPH-related molecules

A second transcriptional control layer governs whether cells can metabolically buffer cystine-derived disulfide stress, primarily by regulating glucose uptake, PPP flux and NADPH-dependent reducing systems. Since disulfidptosis is triggered when the reducing capacity needed to process imported cystine becomes insufficient, transcriptional programs that enhance GLUT1-dependent glucose uptake, PPP activity, or redox-buffering systems can suppress disulfidptosis, whereas disruption of these programs lowers the threshold for lethal disulfide accumulation.

GLUT1, encoded by SLC2A1, is a major facilitative glucose transporter that sustains basal glucose uptake and downstream PPP activity [[67], [68], [69]]. In pancreatic ductal adenocarcinoma (PDAC), the long non-coding RNA (lncRNA) cancer susceptibility 8 (CASC8) represents one of the best-supported upstream suppressors of disulfidptosis [70]. Located at the cancer-associated 8q24 susceptibility locus, CASC8 has been implicated in malignant progression across multiple tumor types [[71], [72], [73]]. CASC8 is preferentially expressed in SLC7A11-high PDAC cells and binds cellular myelocytomatosis viral oncogene homolog (c-Myc), enhancing its protein stability. Through this mechanism, CASC8 activates the c-Myc/GLUT1/PPP pathway, lowers the NADP^+^/NADPH ratio, and preserves reductive buffering capacity. Functionally, CASC8 suppresses glucose starvation-induced disulfide accumulation, protects the actin cytoskeleton from collapse, and antagonizes disulfidptosis. Importantly, this phenotype can be reversed by disulfide-reducing agents such as dithiothreitol (DTT) [74] or tris(2-carboxyethyl)phosphine (TCEP) [75], and can be further potentiated by GLUT1 inhibition with BAY-876. Alternative cell death programs were excluded [70]. These observations place the CASC8/c-Myc/PPP axis among the strongest mechanistic examples linking transcriptional control to metabolic execution of disulfidptosis [70]. Additional transcriptional mechanisms regulate this same metabolic arm through glucose transport. In pancreatic cancer, the lncRNA transmembrane protein 105 (TMEM105) maintains β-catenin stability, activates c-Myc, and promotes GLUT1 transcription, enhancing glucose uptake and PPP-driven NADPH generation [76]. This protects cells against disulfide stress and actin cytoskeletal injury [76]. Similarly, in endometrial cancer, lncRNA EMSLR enhances c-Myc protein stability and nuclear translocation, thus activating the c-Myc/GLUT1 pathway [77]; EMSLR knockdown increases cell death and cytoskeletal collapse, and this phenotype can be rescued by 2-mercaptoethanol (2 ME) [77]. These studies broaden the transcriptional landscape of disulfidptosis from direct control of cystine transport to glucose transport-dependent maintenance of reducing power.

A comparable logic has been observed in head and neck squamous cell carcinoma (HNSCC), where the lncRNA ALMS1 intronic transcript 1 (ALMS1-IT1) acts as a robust negative regulator of disulfidptosis [78]. ALMS1-IT1 is an intronic long noncoding RNA derived from the ALMS1 locus and has been reported to be aberrantly expressed in several malignancies, where it is associated with aggressive behavior and poor clinical outcome [[79], [80], [81]]. In SLC7A11-high HNSCC cells, ALMS1-IT1 positively maintains the transcriptional activity of key PPP enzyme genes, including G6PD, Transketolase (TKT), Phosphoribosyl pyrophosphate synthetase 1 (PRPS1), and Ribokinase (RBKS), at the mRNA level, thereby sustaining intracellular NADPH production. Under glucose starvation, ALMS1-IT1 knockdown increases the NADP^+^/NADPH ratio, promotes disulfide bond formation among actin cytoskeletal proteins, induces filamentous actin (F-actin) collapse, and triggers disulfidptosis. These effects are enhanced by GLUT inhibitors such as BAY-876, and can be rescued by TCEP or by interventions that restore NADPH production [78]. Together, these data support ALMS1-IT1 as a strong transcriptional regulator connecting redox adaptation to the metabolic threshold of disulfidptosis [78].

Beyond endogenous genetic regulators, pharmacological epigenetic modulators can also reshape disulfidptotic susceptibility by altering transcription of NADPH-generating enzymes. Valproic acid (VPA), a widely used antiepileptic drug and potent class I/II histone deacetylase (HDAC) inhibitor, is predicted by network pharmacology and multi-omics analyses to target iron transport, glucose homeostasis, and programmed cell death pathways closely involved in HCC progression [82,83]. VPA suppresses glucose deprivation-induced disulfidptosis through activation of an NRF2-G6PD pathway (comprising nuclear factor erythroid 2-related factor 2 and glucose-6-phosphate dehydrogenase) [83]. Mechanistically, VPA enhances NRF2 binding to the G6PD promoter, increases G6PD expression, elevates PPP-derived NADPH and glutathione levels, reduces aberrant disulfide accumulation, and preserves F-actin integrity under low-glucose stress. Genetic disruption of NRF2 or knockdown of G6PD abolishes this protective effect, strongly supporting a causal role for this redox-buffering axis [83]. Notably, the same study showed that VPA exerts the opposite effect on ferroptosis, sensitizing HCC cells to erastin, sorafenib, and RSL3 by promoting ferritinophagy, increasing labile iron, and enhancing lipid peroxidation independently of the canonical GPX4/SLC7A11 pathway [83,84]. These findings identify G6PD-dependent reductive buffering as a pharmacologically tractable checkpoint of disulfidptotic susceptibility and further illustrate that disulfidptosis and ferroptosis can be divergently regulated within the same tumor context.

#### 5.2.3 Cytoskeleton-related molecules

Beyond cystine loading and reductive buffering, transcriptional programs can also influence whether disulfide stress is translated into the characteristic cytoskeletal collapse of disulfidptosis. The transcriptional layer concerns genes that directly influence the actin cytoskeleton and its associated remodeling machinery, which mediate the structural execution of disulfidptosis. Although cystine overload and NADPH depletion define the metabolic basis of this death program, the final irreversible step involves disulfide-dependent collapse of actin cytoskeletal integrity. Accordingly, transcriptional or signaling programs that regulate actin-remodeling factors can strongly influence the efficiency with which metabolic stress is translated into structural destruction. Evidence for this principle is reinforced by studies of thioredoxin reductase 1 (TrxR1) inhibition in TAZ-activated glioblastoma. TAZ (also known as WWTR1) functions as a transcriptional coactivator that directly drives the expression of TrxR1, leading to marked upregulation of TrxR1, particularly in glucose-deprived 3D tumor conditions via NRF2 signaling [85]. When TrxR1 is suppressed under glucose starvation, intracellular disulfide accumulation increases and a downstream execution program characterized by actin cytoskeleton collapse, peroxisome accumulation, and plasma membrane rupture is triggered [85]. Mechanistically, this process depends on NCKAP1/WAVE complex-mediated cytoskeletal remodeling, thereby linking redox failure to a defined actin-remodeling module. Importantly, the resulting cell death is independent of apoptosis, ferroptosis, and necroptosis, and can be reversed only by disulfide-reducing agents, supporting its classification as bona fide disulfidptosis [85]. These observations indicate that once reductive buffering fails, the transcriptional and signaling state of cytoskeleton-associated effectors can determine how efficiently disulfide stress is converted into lethal structural collapse. Conceptually, this layer governs the execution phase of disulfidptosis. It is mechanistically distinct from SLC7A11-mediated cystine loading and PPP-mediated redox buffering, but functionally inseparable from them: once disulfide stress exceeds metabolic buffering capacity, susceptibility ultimately depends on whether cytoskeletal remodeling machinery permits or amplifies collapse of the actin network [85]. These observations support the view that cytoskeletal regulators are not merely downstream bystanders but active determinants of disulfidptotic execution once redox buffering has failed. At present, however, this branch remains less extensively mapped than the SLC7A11 and NADPH-buffering arms, and many cytoskeletal regulators still await direct transcriptional validation in multiple models.

Related evidence has also emerged in ischemic stroke, where ubiquitin-specific peptidase 15 (USP15) stabilizes the histone methyltransferase SET domain containing 1B (SETD1B) through deubiquitination, thereby increasing H3K4me3 enrichment at the promoters of NCK associated protein 1 like (NCKAP1L) and WASP-family verprolin homologous protein 2 (WAVE2), two actin-regulatory components [86]. USP15 is a deubiquitinating enzyme involved in protein stability control and signal transduction, whereas SETD1B is a COMPASS-family histone methyltransferase that catalyzes H3K4 methylation and promotes transcriptionally active chromatin states [[87], [88], [89], [90]]. Activation of this USP15/SETD1B/NCKAP1L/WAVE2 axis promotes disulfide accumulation and cytoskeleton membrane detachment, thereby facilitating disulfidptosis in brain cells [86]. Such findings provide valuable mechanistic insights and demonstrate that protein stability regulation can converge on actin remodeling machinery to drive the structural disruption observed during disulfidptosis.

Taken together, these studies show that transcriptional regulation of GLUT1, G6PD, PPP-associated genes, c-Myc-driven metabolic programs, and TrxR1-centered redox buffering governs the metabolic tolerance phase of disulfidptosis. This layer determines whether the reducing burden imposed by cystine uptake can still be buffered or instead progresses toward catastrophic NADPH exhaustion. In other words, whereas SLC7A11-related transcriptional regulation imposes cystine metabolic pressure on cells, this layer determines whether such a burden remains metabolically manageable.

### 5.3 Post-transcriptional and protein-stability regulation of the disulfidptotic network

Beyond transcriptional control, disulfidptotic susceptibility can also be shaped at the levels of RNA modification, noncoding RNA-dependent post-transcriptional regulation, ubiquitination/deubiquitination, and protein stability. These mechanisms can rapidly alter the abundance or activity of regulators involved in cystine transport, glucose-redox metabolism, and actin cytoskeletal stability, thereby fine-tuning the threshold and execution of disulfidptosis. As in the transcriptional section above, current evidence can be organized into three functional categories: direct regulation of SLC7A11, direct regulation of PPP- and NADPH-related molecules, and direct regulation of cytoskeleton-related molecules. This parallel structure helps clarify that post-transcriptional regulation does not create an independent disulfidptotic logic, but rather re-tunes the same three core determinants through faster and often more stress-responsive mechanisms.

#### 5.3.1 SLC7A11

A representative example of post-transcriptional regulation has been described in uveal melanoma (UM). Fat mass and obesity-associated protein (FTO), the first characterized N6-methyladenosine (m6A) RNA demethylase, plays a central role in dynamic RNA methylation and has emerged as an important regulator of cancer cell stemness, metabolic plasticity, and therapeutic resistance [[91], [92], [93]]**.** In UM, m6A demethylase FTO is aberrantly upregulated and correlates with reduced global m6A abundance, enhanced malignant behavior, and poor prognosis [94]. Mechanistically, pharmacological or genetic inhibition of FTO increases m6A modification and upregulates SLC7A11 expression, increasing cystine-related redox burden and predisposing cells to disulfidptosis under oxidative stress conditions. In this setting, pharmacologic FTO blockade with meclofenamic acid (MA) depletes glutathione (GSH) and NADPH, induces aberrant disulfide accumulation, and triggers disulfidptotic death that is distinguishable from apoptosis and ferroptosis. This study extends the regulatory landscape of disulfidptosis beyond conventional transcriptional control, and identifies the FTO/m6A/SLC7A11 axis as a functionally relevant epitranscriptomic node linking RNA modification to disulfide-stress vulnerability [94].

#### 5.3.2 PPP/NADPH metabolism and cytoskeletal components

Current understanding of post-transcriptional regulation and protein-stability control governing PPP, NADPH-related molecules, and cytoskeletal components remains restricted to bioinformatic prediction and correlative analysis, with few direct mechanistic investigations.

For example, in sepsis, core disulfidptosis genes including GLUT and cytoskeleton-associated molecules are implicated in PPP flux, NADPH homeostasis, and the maintenance of actin cytoskeletal integrity. Bioinformatic mRNA-miRNA networks imply post-transcriptional repression of these genes, yet such regulation remains experimentally unconfirmed [95]. In autism spectrum disorder (ASD), increased SLC7A11 expression parallels GLUT2 downregulation, NADPH depletion, and altered expression of cytoskeletal proteins including NCKAP1, FLNA, and FLNB. Computational transcription factor-miRNA-mRNA networks further support post-transcriptional silencing of these disulfidptosis regulators, but lack functional validation [96]. In pulmonary arterial hypertension (PAH), the deubiquitinase USP32 and ZNF655 are identified as critical regulators. Predicted ceRNA crosstalk and ubiquitin-related pathways indicate stability modulation of GLUT family proteins, NCKAP1, and WAVE2, without direct experimental support [97].

Collectively, these studies point to miRNA-mediated post-transcriptional control and ubiquitination/deubiquitination-governed protein stability as key uncharacterized mechanisms regulating PPP-NADPH metabolism and cytoskeletal components. However, no direct post-transcriptional targeting events, physiologically relevant protein-stability substrates, or causal links to PPP activity, NADPH levels, and cytoskeletal component function have been verified. Further mechanistic studies are therefore needed to move beyond correlative bioinformatics.

### 5.4 Metabolic buffering and execution machinery of disulfidptosis

The final execution of disulfidptosis is determined by whether cells can sustain sufficient reducing power to detoxify the burden generated by continuous cystine uptake. Accordingly, many regulators act by modulating glycogen mobilization, glucose metabolism, and NADPH homeostasis to govern disulfidptosis progression. In contrast to Sections 4.2, 4.3, which focus on regulatory inputs, this section emphasizes the broader metabolic state that decides whether those inputs culminate in reductive collapse.

A prominent example is the α-KG/TET/YBX1/AMPK pathway (comprising α-ketoglutarate, ten-eleven translocation methylcytosine dioxygenase, Y-box binding protein 1, and AMP-activated protein kinase) identified in HCC and lung cancer models [98]. α-Ketoglutarate (α-KG) is a central tricarboxylic acid (TCA) cycle intermediate that also serves as an essential cofactor for multiple dioxygenases, including TET DNA demethylases, thereby linking mitochondrial metabolism to epigenetic regulation and cell-state control [[99], [100], [101]]. α-KG sustains TET-dependent transcription of YBX1, which in turn promotes AMPK expression and coordinates fatty acid oxidation with the TCA cycle to maintain cytosolic NADPH homeostasis. α-KG depletion, or competition by metabolites such as succinate or itaconate, suppresses this pathway, causing disulfide stress accumulation, actin cytoskeleton disruption, and disulfidptosis [98]. The phenotype can be rescued by exogenous α-KG, AMPK overexpression, or TCEP, while apoptosis and ferroptosis were excluded. This work highlights how central carbon metabolism and epigenetic-metabolic crosstalk can directly determine disulfidptotic execution.

In esophageal squamous cell carcinoma (ESCC), human papillomavirus 16 E6 and E7 proteins (HPV16 E6/E7) promote de novo fatty acid synthesis and thereby consumes large amounts of NADPH [102]. Glucose-6-phosphate dehydrogenase (G6PD) is the rate-limiting enzyme of the oxidative PPP and a major cellular source of NADPH required for redox homeostasis and biosynthetic metabolism [[103], [104], [105]]. Following radiotherapy, this metabolic burden further aggravates cystine-associated disulfide stress and triggers F-actin collapse-mediated disulfidptosis, increasing radiosensitivity. Pharmacological inhibition of G6PD strengthens this effect, whereas inhibitors of apoptosis and ferroptosis do not rescue it [102]. This study is important because it demonstrates that disulfidptosis can be induced not only by reduced glucose uptake but also by excessive NADPH consumption.

Consistent with this principle, GYS1 has emerged as an important metabolic determinant in multiple settings. In TNBC, targeting GYS1 induces abnormal disulfide stress, triggers F-actin contraction, and causes disulfidptosis; the phenotype is reversed by DTT or 2 ME, and alternative death pathways such as ferroptosis or autophagy were excluded [106,107]. These observations reinforce the concept that glycogen metabolism can critically determine whether intracellular glucose reserves are available to sustain PPP-derived NADPH buffering during metabolic stress [108]. In osteoarthritis (OA), chondrocyte disulfidptosis has been proposed to be cooperatively induced by elevated SLC7A11 expression and glucose starvation, which concurrently suppresses GYS1 expression. Current evidence suggests that the GYS1/CCND1/NOD2 axis (comprising glycogen synthase 1, cyclin D1, and nucleotide binding oligomerization domain containing 2) and solute carrier family 2 member 3 (SLC2A3)-dependent glucose metabolism may influence disulfide stress under low-glucose conditions in chondrocytes, but direct structural evidence for bona fide disulfidptosis remains insufficient [109,110].

In ovarian clear cell carcinoma (OCCC), tumor cells naturally possess high glycogen storage, and platinum resistance is driven by the p53-GYS1 positive feedback circuit coupled with glycogen metabolic reprogramming [108]. Wild-type p53 is activated by cisplatin and transcriptionally induces Ring Finger Protein 144a (RNF144a) to degrade GYS1, while GYS1 competitively binds USP14 to promote p53 ubiquitination and degradation, forming a mutual inhibitory loop [108]. Meanwhile, downregulation of GYS1 leads to sustained elevation of p53, which further promotes the transcription and expression of Glycogen Phosphorylase Liver Isoform (PYGL). PYGL is a key rate-limiting enzyme that catalyzes the breakdown of glycogen into glucose-6-phosphate [108]. Under platinum stress, this circuit triggers rapid glycogen mobilization to fuel the pentose phosphate pathway (PPP) for abundant NADPH production. Sustained NADPH generation maintains GSH pools, eliminates ROS, and reduces excessive disulfide bond accumulation, thereby protecting cancer cells from disulfidptosis and ultimately conferring platinum resistance [108].

Additional metabolic regulators appear to operate through related logic. In lung adenocarcinoma (LUAD), Zic family member 5 (ZIC5) supports glycolysis, and its silencing decreases glucose uptake, lactate production, adenosine triphosphate (ATP), NADPH, and GSH, while increasing disulfide stress and cell death that can be partially rescued by TCEP [111]. These studies support the concept that metabolic buffering is central to disulfidptosis, although some are supported by still-developing evidence. Taken together, these findings underscore that the decisive variable is not merely the presence of cystine import, but whether the broader metabolic network can continue to supply reducing equivalents once stress intensifies.

### 5.5 Other regulatory mechanisms

#### 5.5.1 Protein-protein interaction and enzyme activity inhibition

In tumor-infiltrating CD8^+^ T cells, lactate dehydrogenase B (LDHB) interacts with G6PD and suppresses its activity, leading to NADPH depletion, cystine-associated disulfide stress, actin cytoskeleton injury, and T-cell exhaustion [112]. This process depends on SLC7A11-mediated cystine uptake and can be reversed by reducing agents or by limiting cystine transport [112]. These findings are especially significant because they suggest that disulfidptosis may contribute not only to tumor cell death but also to dysfunction of anti-tumor immunity.

#### 5.5.2 Membrane trafficking/endosomal recycling

In epileptic neurons, seizure activity downregulates RAS oncogene family-like 11A (Rab11a). Rab11a is a small GTPase that regulates recycling endosome trafficking and is required for efficient return of membrane proteins to the cell surface [[113], [114], [115]]. Loss of Rab11a impairs recycling of glucose transporter 3 (GLUT3) to the plasma membrane, thereby reducing glucose uptake, depleting NADPH, and promoting cystine-associated disulfide accumulation [116]. Rab11a overexpression restores GLUT3 recycling, improves glucose uptake, and maintains the NADP^+^/NADPH balance [116]. However, because this study did not fully document canonical structural hallmarks such as actin disulfide crosslinking or rigorously exclude competing death pathways in vivo, the evidence is better interpreted as partial rather than definitive support for bona fide disulfidptosis.

### 5.6 Summary of the regulatory landscape

**Fig. 3:**
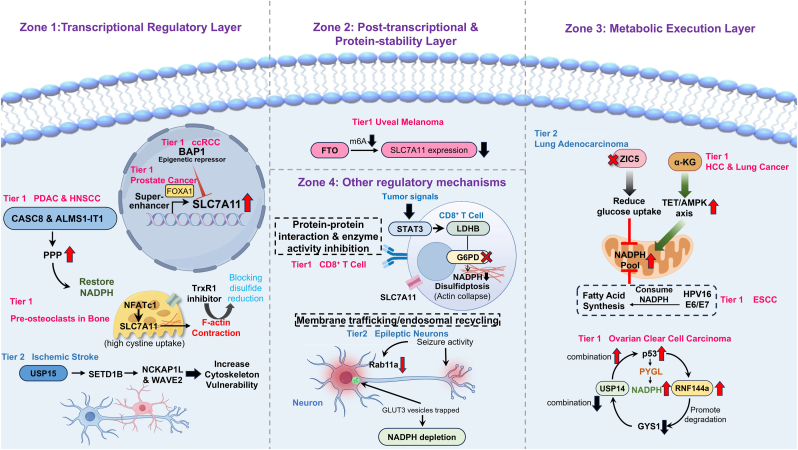
Multi-layer regulatory landscape governing disulfidptotic susceptibility.

With the expansion of disulfidptosis research, validated regulators must be distinguished from indirect or associated factors. Robust evidence requires dependence on SLC7A11, NADPH depletion, actin disulfide accumulation, F-actin collapse, and rescue by reducing agents. Because metabolic perturbation in vivo may engage mixed cell death phenotypes, disulfidptosis is most rigorously defined when disulfide stress acts as the dominant driver of actin cytoskeletal collapse. Strict evidentiary criteria are therefore essential to establishing disulfidptosis as a robust framework for precision metabolic intervention (Fig. 3, Table 2).

**Table 2:** Representative regulators of disulfidptosis across different evidence tiers.

Tier	Disease/Model	Regulator	Mechanistic rationale for tier assignment
Tier 1	Pancreatic ductal adenocarcinoma	CASC8	Strong mechanistic evidence. CASC8 binds c-Myc, enhances pentose phosphate pathway activity, sustains NADPH production, and limits glucose deprivation-induced disulfide stress, actin cytoskeleton collapse, and disulfidptosis. The phenotype is modulated by metabolic intervention and reducing conditions [70].
Tier 1	Uveal melanoma	FTO	FTO is highly expressed in uveal melanoma and is associated with reduced m6A modification, aggressive behavior, and poor prognosis. Pharmacologic inhibition of FTO restores m6A levels, upregulates SLC7A11, depletes GSH and NADPH, and promotes abnormal disulfide accumulation, thereby inducing disulfidptosis-related death. A nanodrug formulation delivering the FTO inhibitor further suppresses tumor growth and enhances antitumor efficacy in vivo [94].
Tier 1	Head and neck squamous cell carcinoma	ALMS1-IT1	Strong mechanistic evidence. In SLC7A11-high HNSCC cells, ALMS1-IT1 supports PPP-related metabolic activity and NADPH homeostasis. Its silencing under glucose starvation increases oxidative/disulfide burden, promotes cytoskeletal damage, and enhances disulfidptosis-like death, which is attenuated by reducing treatment or restoration of redox balance [78].
Tier 1	Hepatocellular carcinoma and lung cancer	α-KG/TET/YBX1/AMPK axis	Strong mechanistic evidence. This axis supports cytosolic NADPH homeostasis. α-KG depletion increases disulfide stress, disrupts the actin cytoskeleton, and promotes disulfidptosis-related death, whereas α-KG supplementation, AMPK restoration, or reducing treatment alleviates the phenotype [98].
Tier 1	Clear cell renal cell carcinoma	BAP1	Strong mechanistic evidence. BAP1 represses SLC7A11 expression, limits cystine uptake, and reduces susceptibility to glucose starvation-induced disulfide stress and cytoskeletal injury in a deubiquitinase-dependent manner. Genetic or pharmacologic suppression of SLC7A11 diminishes this vulnerability [50].
Tier 1	Prostate cancer	Super-enhancer/FOXA1/SLC7A11 axis	Strong mechanistic evidence. A super-enhancer-driven FOXA1 program activates SLC7A11 transcription. Under glucose limitation or GLUT1 inhibition, this axis increases cystine uptake-dependent disulfide stress, promotes actin cytoskeleton collapse, and facilitates disulfidptosis-related death [55].
Tier 1	Pancreatic cancer	TMEM105/β-catenin/c-MYC/GLUT1 axis	Strong mechanistic evidence. TMEM105 stabilizes β-catenin, activates c-MYC, and increases GLUT1 expression, thereby promoting glucose uptake and PPP-associated NADPH generation, relieving disulfide stress, and limiting cytoskeletal injury [76].
Tier 1	Endometrial carcinoma	EMSLR	Strong mechanistic evidence. EMSLR regulates the c-MYC/GLUT1 pathway to sustain glucose utilization and NADPH production. Its knockdown enhances glucose deprivation-induced cytoskeletal damage and cell death, and the phenotype is alleviated by reducing treatment [77].
Tier 1	Esophageal squamous cell carcinoma	HPV16 E6/E7/G6PD axis	Strong mechanistic evidence. HPV16 E6/E7-driven metabolic reprogramming increases NADPH consumption, and radiotherapy further aggravates cystine-associated disulfide stress and actin damage. G6PD inhibition strengthens this phenotype, supporting a disulfidptosis-related mechanism [102].
Tier 1	Ovarian clear cell carcinoma	p53/GYS1 circuit	Strong mechanistic evidence. This regulatory circuit mobilizes glycogen into the PPP, increases NADPH production, alleviates disulfide stress, preserves actin cytoskeletal integrity, and suppresses disulfidptosis-related death during cisplatin exposure [108].
Tier 1	Triple-negative breast cancer	GYS1	Strong mechanistic evidence. GYS1 targeting induces disulfide stress, F-actin contraction, and disulfidptosis-related death, and the phenotype is reduced by thiol-based reducing agents. Alternative death pathways such as ferroptosis or autophagy were examined in the original study [106].
Tier 1	Pre-osteoclasts	NFATc1/SLC7A11/TRXR1 axis	Strong mechanistic evidence. NFATc1 transcriptionally activates SLC7A11, whereas TRXR1 inhibition impairs disulfide reduction, leading to cystine accumulation, disulfide stress, F-actin contraction, and disulfidptosis-related death. The phenotype can be rescued by SLC7A11 inhibition or disulfide-reducing agents [65].
Tier 1	Tumor-infiltrating CD8^+^ T cells	LDHB/G6PD axis	Strong mechanistic evidence. LDHB interacts with G6PD and suppresses its activity, decreases NADPH availability, promotes cystine accumulation and disulfide stress, and contributes to actin cytoskeletal injury and exhaustion-associated disulfidptosis-related death in tumor-infiltrating CD8^+^ T cells [112].
Tier 1	Glioblastoma	TAZ/TrxR1/NCKAP1-WAVE axis	Strong mechanistic evidence. In TAZ-activated glioblastoma, TrxR1 suppression under glucose starvation increases intracellular disulfide accumulation and triggers actin cytoskeleton collapse through NCKAP1/WAVE complex-associated remodeling. Cell death is independent of apoptosis, ferroptosis, and necroptosis and is reversed by disulfide-reducing agents [85].
Tier 2	Colorectal cancer	DPP7/GPX4 axis	Mechanistically supportive but not fully pathway-definitive evidence. DPP7 stabilizes GPX4 and helps preserve redox balance under metabolic stress. DPP7 depletion increases disulfide bond formation in actin cytoskeleton-associated proteins and promotes F-actin remodeling or collapse. However, because this pathway is closely connected to GPX4-mediated antioxidant regulation, further work is needed to separate canonical disulfidptosis from broader redox stress responses [117].
Tier 2	Ischemic stroke	USP15/SETD1B/NCKAP1L/WAVE2 axis	Mechanistically developed but still not fully definitive evidence. USP15 stabilizes SETD1B and promotes transcriptional activation of NCKAP1L and WAVE2, enhancing abnormal disulfide accumulation and cytoskeleton-membrane detachment consistent with disulfidptosis-related injury. Full canonical structural validation and exclusion criteria remain less complete than in optimized cell models [86].
Tier 2	Epilepsy (neuronal model)	Rab11a/Glut3 axis	Partial mechanistic support. Rab11a downregulation impairs GLUT3 recycling, reduces glucose uptake and NADPH availability, increases disulfide burden, and induces neuronal disulfidptosis-related death. However, the full canonical structural and exclusion criteria are not yet equally well established in vivo [116].
Tier 2	Hepatocellular carcinoma	CD2AP	Partial mechanistic support. CD2AP appears to help maintain NADPH homeostasis and reduce sensitivity to glucose starvation-associated disulfide stress, but the direct execution link to actin collapse and canonical disulfidptosis remains incompletely defined [118,119].
Tier 2	Breast cancer	circ_0022382	Partial mechanistic support. circ_0022382 activates PI3K/AKT/mTOR signaling and upregulates SLC7A11, thereby supporting cystine uptake and redox buffering against glucose starvation-associated injury. However, direct evidence for terminal cytoskeletal execution remains limited [66].
Tier 2	Pancreatic adenocarcinoma	G6PD/SLC7A11 axis	Partial mechanistic support. G6PD supports NADPH production and redox homeostasis in PAAD. Its inhibition promotes cytoskeletal contraction and cell death under metabolic stress, but direct delineation of the full disulfidptosis execution cascade remains incomplete [120].
Tier 2	Lung adenocarcinoma	ZIC5	Partial mechanistic support. ZIC5 silencing suppresses glycolytic activity, decreases glucose uptake, lactate production, ATP, NADPH, and GSH, and increases disulfide stress-associated cell death. However, direct evidence for actin-centered terminal execution and comprehensive exclusion of alternative death pathways remain limited [111].
Tier 2	Osteoarthritis	GYS1/CCND1/NOD2 axis	Partial mechanistic support. Under low-glucose conditions, GYS1 downregulation is associated with enhanced disulfide stress and cartilage degeneration through the CCND1/NOD2 axis, but direct evidence for canonical disulfidptosis execution remains insufficient [110].
Tier 2	Head and neck squamous cell carcinoma	THBS1	Partial mechanistic support. THBS1 has been linked to NADPH redox balance, disulfide stress, F-actin contraction, and modulation of SLC7A11-related phenotypes under glucose deprivation. However, direct demonstrations of actin disulfide crosslinking, canonical rescue, and exclusion of competing pathways remain less comprehensive than for ALMS1-IT1 [153].
Tier 3	Autism spectrum disorder	SLC7A11-related network	Association-based or partial network-level support. A network centered on SLC7A11, altered glucose transport, NADPH depletion, and cytoskeletal genes suggests possible involvement of disulfide stress-related injury, but direct evidence for actin cytoskeleton collapse and bona fide disulfidptosis is lacking [96].
Tier 3	Neuroblastoma	TFAP2D	Association-based evidence. TFAP2D is identified as a disulfidptosis- and fatty acid metabolism-related factor associated with poor prognosis and malignant progression, but direct mechanistic validation of disulfidptosis is unavailable [121].
Tier 3	Gastric cancer	GAMT	Association-based evidence. GAMT is identified in disulfidptosis-related transcriptomic subtypes of gastric cancer, but no direct evidence links it to disulfide stress-driven cytoskeletal death [122].
Tier 3	Ulcerative colitis	SLC3A2	Association-based evidence. SLC3A2 is associated with intestinal inflammation, disease course, immune features, and disulfidptosis-related gene programs, but current support for a direct role in disulfidptosis is correlative [123].
Tier 3	Osteosarcoma	LINC01137	Association-based evidence. LINC01137 is identified as a disulfidptosis-related lncRNA associated with proliferation, invasion, macrophage polarization, and immune remodeling, but direct mechanistic evidence is lacking [124].
Tier 3	Osteosarcoma	PABPC3	Association-based evidence. PABPC3 is linked to progression and immune suppression in osteosarcoma as a disulfidptosis-related risk gene, but core mechanistic events have not been validated [125].
Tier 3	Parkinson's disease	IQGAP1	Association-based evidence. IQGAP1 is associated with Parkinson's disease and cytoskeletal remodeling as a disulfidptosis-related diagnostic biomarker, but current support for direct involvement in disulfidptosis remains indirect [126].
Tier 3	Osteoarthritis	SLC2A3	Association-based evidence. SLC2A3 is linked to osteoarthritis and disulfidptosis-related signatures, but mechanistic validation remains insufficient [109].

**Tier definition:** Tier 1 indicates strong mechanistic evidence linking the regulator to key features of disulfidptosis, including metabolic dependence, disulfide stress, and cytoskeletal injury. Tier 2 indicates partial mechanistic support, with some but not all core features experimentally addressed. Tier 3 indicates association-based evidence derived mainly from transcriptomic, prognostic, or bioinformatic analyses without direct mechanistic validation. Tier assignment was interpretive and narrative rather than statistically weighted; it was based on mechanistic convergence across reported experiments.

Taken together, this section systematically outlines a layered regulatory network of disulfidptosis centered on cystine transport, maintenance of NADPH redox homeostasis and actin cytoskeletal damage. Although reductionist mechanistic studies have clarified key molecular events in disulfidptosis, they are less suited to explaining tissue- and disease-specific heterogeneity in susceptibility or to identifying potential novel regulators through large-scale data integration. Integrated multi-omics strategies provide a feasible approach to overcoming these limitations.

Disulfidptosis is controlled by an integrated regulatory network organized here into three major layers. The transcriptional layer includes regulators that determine cystine loading or reductive buffering capacity, such as BAP1, FOXA1-driven super-enhancer signaling, NFATc1, CASC8, ALMS1-IT1, and the USP15-SETD1B axis. The post-transcriptional and protein-stability layer includes epitranscriptomic and proteostatic mechanisms that modulate vulnerability, exemplified by FTO-dependent regulation of SLC7A11. The metabolic buffering and execution layer comprises pathways that determine whether cystine-derived disulfide stress can be neutralized or instead converted into structural collapse, including the p53/GYS1/PYGL circuit, α-KG/TET/AMPK signaling, HPV16 E6/E7-driven fatty acid synthesis, and silencing of ZIC5 leads to reduced glucose uptake. Other regulatory mechanisms shown in the figure include LDHB-mediated inhibition of G6PD and Rab11a-dependent GLUT3 recycling. Together, these regulatory inputs converge on three core determinants of disulfidptosis: cystine uptake, NADPH availability, and actin-cytoskeletal susceptibility to disulfide stress.

## 6 Multi-omics prediction and validation of disulfidptosis susceptibility

### 6.1 Current status and unmet need

Current studies on disulfidptosis include both mechanistic investigations and bioinformatic analyses, but multi-omics evidence has so far been discussed only in a fragmented manner. The rapid emergence of disulfidptosis-related prognostic signatures and molecular subtypes highlights the potential of computational stratification, but also raises the need for cautious interpretation [106,127,128]. Most available models rely predominantly on transcriptomic signatures, differential expression analysis, or prognosis-related coefficients, whereas systematic evaluation across genomic, epigenomic, proteomic, metabolomic, single-cell, and spatially resolved layers remains limited [129,130]. Such signatures may capture SLC7A11-high states, redox-related transcriptional programs, or general disease aggressiveness, but they do not necessarily indicate functional susceptibility to disulfidptosis. As a result, current predictions often capture association more readily than mechanism, and their biological specificity for disulfidptosis requires careful validation. An integrated framework is therefore needed to define the predictive value, biological scope, and practical limitations of multi-omics approaches in this field.

### 6.2 Predictive value and limitations of different omics layers

Different omics layers provide complementary but non-equivalent information for predicting disulfidptosis susceptibility [131,132]. Genomic and epigenomic data can reveal relatively stable upstream determinants, including copy-number variation, mutation patterns, chromatin accessibility, and DNA methylation states that may influence cystine transport, antioxidant capacity, or redox regulation [131]. Transcriptomic data are well suited for large-scale stratification and hypothesis generation, particularly for identifying SLC7A11-associated programs, cysteine metabolism, glutathione synthesis, NADPH-producing pathways, and cytoskeletal regulators [133]. However, mRNA abundance alone does not necessarily reflect protein activity, metabolite availability, or execution competence [134]. Proteomic analysis is more informative for downstream effector mechanisms, especially when focused on SLC7A11-related transport programs, redox enzymes, glutathione metabolism, and actin cytoskeletal regulators [135,136]. Metabolomic profiling is particularly relevant because disulfidptosis is tightly linked to cystine accumulation, NADPH exhaustion, altered GSH/GSSG balance, and disulfide stress [1,3]. Emerging single-cell and spatially resolved approaches further extend the predictive value of multi-omics analysis. Single-cell RNA sequencing and single-cell multi-omics can resolve intratumoral or tissue-level heterogeneity that is masked in bulk datasets, thereby identifying rare cell populations or cell states characterized by high SLC7A11 expression, altered cysteine metabolism, impaired NADPH-generating programs, or cytoskeletal vulnerability [137,138]. These approaches may be particularly useful for distinguishing whether a disulfidptosis-related signature reflects tumor-intrinsic susceptibility or signals derived from stromal, immune, or other microenvironmental compartments [139,140]. Spatial transcriptomics can further preserve tissue architecture and reveal whether putatively susceptible cells are located within metabolically relevant niches, such as regions with limited glucose availability, hypoxia, vascular heterogeneity, or strong interactions with the tumor microenvironment [141,142]. Spatial proteomics, including multiplexed imaging-based platforms, may provide more direct information on protein-level redox regulators, SLC7A11-associated programs, and actin cytoskeletal remodeling in situ. Nevertheless, no single omics layer is sufficient on its own. Upstream genomic or transcriptional features may not predict whether cells can execute disulfidptosis, whereas proteomic and metabolomic states, although mechanistically closer to the death process, are often more context dependent and less readily available in large clinical cohorts. Similarly, single-cell or spatial enrichment of SLC7A11, redox-related transcripts, or prognostic risk scores should not be interpreted as definitive evidence of disulfidptosis without biochemical readouts and perturbation-based validation.

### 6.3 Integrated prediction and experimental validation

The main value of multi-omics integration lies not only in improving in silico prediction of disulfidptosis susceptibility, but also in prioritizing experimentally testable mechanisms [143,144]. By combining transcriptomic, proteomic, metabolomic, single-cell, and spatially resolved features with pathway annotation and functional network analysis, integrated models may help identify tumors, tissue niches, or cellular states that are more likely to depend on SLC7A11-mediated cystine uptake, become vulnerable under glucose limitation, exhibit impaired NADPH regeneration, or accumulate disulfide stress [3]. At the same time, integrated models should not be interpreted as direct evidence of disulfidptosis unless their predictions are linked to defining biochemical and phenotypic features. Candidate regulators or biomarkers emerging from multi-omics analysis should therefore be validated through genetic or pharmacological perturbation, temporal profiling, and mechanism-relevant rescue experiments. Ideally, validation should assess cystine-loading susceptibility, glucose deprivation-dependent vulnerability, NADPH depletion, actin cytoskeletal disulfide damage, and rescue by interventions that directly target the proposed mechanism, while excluding major competing death programs such as apoptosis, ferroptosis, necroptosis, and cuproptosis. In this way, multi-omics analysis can support a more rigorous workflow from computational discovery to mechanistic confirmation, distinguishing associative biomarkers from regulators that directly govern disulfidptosis susceptibility.

## 7 Clinical pathophysiology: tissue specificity and metabolic heterogeneity

### 7.1 Neoplastic diseases

#### 7.1.1 Gynecologic and breast malignancies

Among gynecologic malignancies, the most compelling evidence currently comes from OCCC and endometrial cancer. By contrast, the evidence in breast cancer is more heterogeneous and therefore requires stratified interpretation according to the strength of mechanistic validation.

In OCCC, cellular tolerance to disulfidptosis is substantially influenced by the p53-GYS1 regulatory loop [108]. This circuit suppresses disulfidptosis triggered by aberrant disulfide accumulation, thereby enhancing tumor cell resistance to cisplatin. In this context, disulfidptosis functions as a form of cell death that can be antagonized by glycogen metabolism and thus represents a key target for reversing platinum resistance in OCCC [108]. This mechanism directly links glycogen metabolism, NADPH maintenance, cytoskeletal protection, and platinum resistance, making it one of the strongest lines of evidence connecting disulfidptosis with chemoresistance in gynecologic malignancies [108].

In endometrial cancer, the lncRNA EMSLR promotes glucose uptake and PPP derived NADPH production by activating the c-Myc/GLUT1 pathway, thereby lowering the NADP^+^/NADPH ratio to alleviate disulfide stress [77]. Through this mechanism, EMSLR suppresses glucose deprivation induced disulfidptosis while promoting tumor proliferation, progression, and chemoresistance [77]. Here, disulfidptosis acts as a tumor-suppressive form of cell death, and its activation can inhibit endometrial cancer development, supporting its potential as a therapeutic target in this disease.

In triple negative breast cancer (TNBC), glycogen synthase 1, GYS1, acts as a negative regulator of disulfidptosis [106]. By promoting glycogen synthesis and accumulation and maintaining NADPH levels, GYS1 counteracts disulfide stress, thereby suppressing disulfidptosis and promoting tumor proliferation and migration [106]. Targeting GYS1 to trigger disulfidptosis therefore represents a highly promising therapeutic strategy. By comparison, in broader breast cancer contexts, the EIF4A3/circ_0022382/let 7a 5p/PI3K/AKT mTOR/SLC7A11 axis currently carries only Tier 2 evidence [66]. Available data support that circ_0022382 upregulates SLC7A11, sustains cystine uptake and NADPH levels, and that its knockdown enhances sensitivity to glucose starvation-induced cell death. However, direct evidence for intracellular disulfide accumulation and actin cytoskeletal disulfide damage remains insufficient [66]. Accordingly, this pathway is better regarded at present as a potential upstream regulatory mechanism of disulfidptosis rather than a fully validated execution pathway.

Overall, in female malignancies, the strongest findings converge on a common principle: metabolic buffering systems that preserve NADPH, whether through glycogenolysis, PPP activation, or regulation of glucose transport, constitute a critical defense against disulfidptosis. This conclusion is supported by Tier 1 studies in OCCC, endometrial cancer, and TNBC, whereas other mechanisms based on noncoding RNAs remain supportive but not yet decisive evidence.

#### 7.1.2 Digestive system malignancies

Among digestive system tumors, pancreatic cancer, hepatocellular carcinoma, and esophageal squamous cell carcinoma currently provide some of the strongest mechanistic evidence for the regulation of disulfidptosis. These tumors commonly grow in nutrient deprived microenvironments and therefore rely heavily on glucose flux, PPP activity, and SLC7A11 dependent cystine metabolism to avoid lethal disulfide stress.

Disulfidptosis is a key metabolically dependent form of programmed cell death in PDAC. It functions as an important tumor-suppressive cell death mechanism that restrains tumor progression, and its activity is tightly suppressed within tumor cells by multiple metabolic regulatory axes. Among these, activation of the PPP through the CASC8/c-Myc/GLUT1 axis can markedly increase NADPH generation and relieve intracellular disulfide stress [70]. At the same time, the β-catenin/c-Myc/GLUT1 signaling axis can be activated under the regulation of lncRNA TMEM105 [76], thereby enhancing cellular glucose uptake and PPP flux, maintaining NADPH redox homeostasis, suppressing glucose starvation triggered disulfidptosis, and conferring greater tolerance to metabolic stress and stronger survival capacity in the nutrient deprived tumor microenvironment, ultimately promoting malignant progression of pancreatic cancer [76]. Collectively, these studies indicate that PDAC can evade disulfidptosis by simultaneously strengthening glucose input and NADPH production. By contrast, G6PD in pancreatic adenocarcinoma (PAAD) currently has only Tier 2 evidence [120]. Although its inhibition reduces NADPH and promotes F-actin contraction, direct evidence for the sequence of disulfide accumulation followed by cytoskeletal damage has not yet been fully established.

In colorectal cancer (CRC), dipeptidyl peptidase 7 (DPP7) has been linked to the regulation of glucose-dependent redox and cytoskeletal stress responses. DPP7, also termed dipeptidyl peptidase II or quiescent cell proline dipeptidase, is an intracellular serine protease involved in post-translational peptide processing and has been associated with lymphocyte viability and cellular homeostasis [145]. In CRC models, DPP7 was reported to interact with and stabilize GPX4, thereby contributing to redox homeostasis under metabolic stress [117]. Under glucose starvation, DPP7 depletion increases cellular susceptibility to disulfide stress and is accompanied by abnormal disulfide bond formation in actin cytoskeleton-associated proteins, including Drebrin, FLNA, and filamin B (FLNB), as well as F-actin remodeling or collapse [117]**.** Restoration of GPX4 partially reverses these phenotypes, and competing death programs such as ferroptosis were examined in the original study [117]. These findings suggest that DPP7 may buffer a glucose-dependent redox-cytoskeletal vulnerability in CRC. However, because this pathway is closely connected to GPX4-mediated antioxidant regulation, further work is needed to determine how specifically DPP7 participates in the canonical disulfidptosis cascade and whether its effects can be separated from broader redox stress-response mechanisms. FLNA has also been implicated in glucose-dependent cytoskeletal adaptation and disulfidptosis-related phenotypes in CRC. FLNA was reported to stabilize the cytoskeleton under glucose-dependent conditions, reduce disulfidptosis-associated cellular changes, and promote tumor progression by shaping an immunosuppressive microenvironment with decreased CD8^+^ T-cell infiltration [146]. Combined treatment with a GLUT1 inhibitor and anti-PD-1 therapy was shown to enhance antitumor effects, suggesting a possible therapeutic connection among glucose dependence, cytoskeletal vulnerability, and immune modulation [146]. Taken together, available CRC studies indicate that DPP7 and FLNA are both associated with glucose-dependent redox imbalance, cytoskeletal remodeling, and disulfidptosis-like cellular phenotypes. Their relevance to CRC progression and therapy response is intriguing, particularly in relation to metabolic stress and immune regulation. Future studies should further validate hallmark events such as disulfide accumulation, actin cytoskeletal protein crosslinking, F-actin collapse, and rescue by thiol-reducing or metabolic interventions to define their precise positions within the disulfidptosis framework.

Disulfidptosis has shown important translational potential in the treatment of hepatocellular carcinoma (HCC), especially drug resistant HCC, and its activity can be regulated by both natural small molecules and targeted small molecule compounds through multiple signaling pathways. The natural small molecule Gaudichaudione H (GH) can activate the NRF2/SLC7A11 signaling axis through an autophagy mediated noncanonical mechanism [147], markedly increasing the sensitivity of HCC cells to glucose-starvation-induced disulfidptosis, and this effect is independent of ferroptosis [147]. GH increases NRF2 protein levels and promotes transcription of its downstream target gene SLC7A11, thereby further aggravating cellular cystine uptake, disrupting GSH synthesis, and depleting nicotinamide adenine dinucleotide phosphate, NADPH, ultimately inducing abnormal disulfide crosslinking of cytoskeletal proteins and triggering disulfidptosis. This effect depends entirely on the functional integrity of NRF2 and SLC7A11, while showing no obvious toxicity toward normal hepatocytes, thus providing a natural product lead for intervention in drug resistant HCC.

At the same time, the mitochondria-targeted small molecule Alexidine dihydrochloride (AD) first induces mitochondrial stress and the mitochondrial unfolded protein response (UPRmt) [148], in HCC cells, thereby activating the ATF4/DDIT3 signaling axis (comprising activating transcription factor 4 and DNA damage-inducible transcript 3) and significantly enhancing disulfidptosis under glucose starvation. This process is independent of SLC7A11 expression and eIF2α phosphorylation [148]. Through the ATF4/DDIT3 pathway, AD disrupts amino acid metabolism, inhibits GSH synthesis, and reduces NADPH levels, thereby aggravating disulfide stress and cytoskeletal collapse. Knockdown of either ATF4 or DDIT3 completely reverses the disulfidptosis promoting effect of AD, identifying the mitochondrial stress/ATF4/DDIT3 axis as a novel pathway regulating disulfidptosis in HCC and providing new molecular targets and intervention strategies for overcoming apoptosis and ferroptosis resistant liver cancer [148]. In addition, the α-KG/TET/YBX1/AMPK axis across HCC and lung cancer models can maintain cytosolic NADPH and prevent actin collapse and disulfidptosis [98]. By contrast, CD2AP in HCC currently supports only a Tier 2 functional role at the levels of NADPH alteration and morphology [118,119], whereas other studies are more associative in nature. For example, in HCC cells with high SLC7A11 expression, overexpression of ribophorin 1 (RPN1) regulates the expression of cell cycle related proteins [149], including cyclin-dependent kinase 1/2 (CDK1/2), Cyclin D1, and Cyclin E1, arrests the cell cycle at G0/G1 phase, and inhibits proliferation and migration. These effects can be enhanced by reducing cystine uptake and restoring redox balance, which to some extent supports an association between RPN1, disulfide stress, and actin cytoskeletal stability [149].

In ESCC, the current evidence is likewise strong. HPV16 E6/E7/G6PD related metabolic reprogramming is supported by Tier 1 evidence. HPV16 drives de novo fatty acid synthesis and thereby consumes NADPH [102]. After radiotherapy, this process further aggravates cystine accumulation, promotes disulfide stress, leads to F-actin collapse, and induces disulfidptosis. This study is especially important because it incorporates viral oncogenesis, radiotherapy response, and disulfidptosis into a single mechanistic framework [102].

Overall, digestive system tumors most clearly demonstrate that maintenance of the PPP, glucose uptake, glycogen utilization, and direct protection of actin associated proteins are core determinants of whether cells can resist disulfidptosis.

#### 7.1.3 Urinary system malignancies

In ccRCC, available evidence indicates that the BAP1/SLC7A11/NADPH axis markedly suppresses glucose starvation induced disulfidptosis by inhibiting SLC7A11 mediated cystine uptake and maintaining a low NADP^+^/NADPH ratio [50]. Nevertheless, current research on disulfidptosis in kidney renal clear cell carcinoma (KIRC) is predominantly based on multi-omics data. Several studies have screened out core regulatory genes including PDLIM1, while other studies have established prognostic models by integrating disulfidptosis with ferroptosis, which provides credible references for prognostic evaluation and targeted therapy of KIRC [[150], [151], [152]].

In prostate cancer, the super-enhancer/FOXA1 axis significantly promotes disulfidptosis by upregulating SLC7A11 expression and aggravating NADPH depletion and aberrant disulfide accumulation under glucose starvation [55]. As a selective lethal mode in high SLC7A11 expressing prostate cancer cells within a glucose deficient microenvironment, disulfidptosis represents a critical metabolic vulnerability in prostate cancer, and targeting this axis or using GLUT1 inhibitors can induce disulfidptosis to suppress tumor progression [55].

In bladder cancer, direct mechanistic evidence for disulfidptosis remains relatively weak. Current studies based on the POU5F1/CTSE axis (comprising POU class 5 homeobox 1 and cathepsin E) and the DRG signature model can only be classified as Tier 3 evidence [153]. The POU5F1/CTSE axis is a key transcriptional regulatory pathway in bladder cancer, in which POU5F1 directly transcriptionally activates CTSE and promotes tumor proliferation and invasion. Although this axis is associated with oxidative stress and metabolic stress, it has not been shown to drive cystine disulfide accumulation and actin collapse. The Disulfidptosis-Related Genes (DRG) signature model can be used for molecular subtyping as well as prediction of prognosis and therapeutic response in bladder cancer. However, it remains an association analysis and does not validate core hallmark events of disulfidptosis such as disulfide overload, NADPH depletion, and cytoskeletal destruction [153].

#### 7.1.4 Lung adenocarcinoma

Compared with digestive or urinary system tumors, the evidentiary landscape of disulfidptosis in lung cancer is more uneven and therefore requires strict discrimination by evidence tier. ZIC5 in LUAD currently has Tier 2 evidence [111]. Existing studies indicate that ZIC5 is associated with the expression of glycolytic genes, and its silencing reduces glucose uptake, lactate production, ATP levels, NADPH, and GSH, while increasing disulfide stress. TCEP can partially rescue this phenotype [111]. However, direct evidence for actin specific disulfide crosslinking remains lacking, and alternative cell death pathways have not been comprehensively excluded. Therefore, ZIC5 is more appropriately described at present as a candidate factor that modulates susceptibility to disulfidptosis rather than a fully established execution factor. Moreover, fibrinogen alpha chain (FGA) has been reported to exert a tumor-suppressive effect by modulating SLC7A11 stability and a disulfidptosis-associated phenotype, supported by Tier 2 evidence [154]. Mechanistically, FGA physically interacts with SLC7A11 and stabilizes the SLC7A11 protein to maintain its expression [154]. In the glucose-deprived tumor microenvironment, FGA-mediated SLC7A11 stabilization promotes cystine uptake and NADPH consumption. According to the core mechanism of disulfidptosis, this is thought to induce aberrant disulfide accumulation, actin cytoskeletal collapse, and disulfidptosis, thereby inhibiting tumor progression.

Beyond this, current research on disulfidptosis in LUAD mainly focuses on DRGs or disulfidptosis-related ferroptosis genes (DFRGs), involving the construction of prognostic models, analysis of immune microenvironment and drug sensitivity, as well as in vitro validation of key genes, but evidence related to disulfidptosis itself is insufficient and only limited to correlation analysis, without in-depth exploration of its specific mechanism in LUAD progression [[155], [156], [157], [158]].

#### 7.1.5 Head and neck squamous cell carcinoma

Among the reported regulators of disulfidptosis in HNSCC, ALMS1-IT1 currently has relatively strong experimental support. ALMS1-IT1 is markedly upregulated in HNSCC cells with high SLC7A11 expression and functions as a negative regulator of disulfidptosis. Mechanistically, ALMS1-IT1 sustains pentose phosphate pathway activity and NADPH production, thereby helping to maintain redox homeostasis under glucose starvation. Silencing ALMS1-IT1 increases the NADP^+^/NADPH ratio, aggravates disulfide stress, promotes abnormal intermolecular disulfide bond formation among actin cytoskeletal proteins, and induces F-actin collapse [78]. In comparison, thrombospondin-1 (THBS1) has been proposed as a potential disulfidptosis-associated regulator in HNSCC, although the current evidence remains less comprehensive [159]. Available data suggest that THBS1 may help maintain intracellular NADPH redox balance, reduce the NADP^+^/NADPH ratio, and alleviate disulfide stress under glucose-deprived conditions. THBS1 has also been reported to mitigate F-actin contraction and cell shrinkage and to modulate the expression of disulfidptosis-related genes, including SLC7A11 [159]. However, compared with ALMS1-IT1, the evidence supporting THBS1 as a direct regulator of canonical disulfidptosis remains relatively limited, particularly with respect to direct demonstrations of actin disulfide crosslinking, F-actin collapse, and rescue by thiol-reducing agents or metabolic interventions [159]. Collectively, current evidence indicates that ALMS1-IT1 is a well-supported negative regulator of disulfidptosis in HNSCC, whereas THBS1 should be regarded as a candidate or moderate-evidence regulator. Both factors may contribute to the resistance of HNSCC cells to glucose starvation-induced disulfide stress by preserving NADPH-dependent redox homeostasis. Further mechanistic studies are required to determine whether candidate regulators directly control the canonical disulfidptosis cascade or instead act indirectly through broader redox and stress-response pathways.

#### 7.1.6 Glioma

Unlike HNSCC, pancreatic cancer, and kidney cancer, where evidence is already relatively substantial, the evidence for disulfidptosis in glioma and glioblastoma is highly uneven. At present, only the induction of disulfidptosis via TrxR1 inhibition combined with glucose deprivation is supported by relatively strong evidence [85]. In highly invasive glioblastoma driven by TAZ, disulfidptosis represents a key metabolic vulnerability that can be precisely triggered by combining TrxR1 inhibitors with glucose deprivation [85]. This strategy can directly kill tumor cells, suppress growth, and prolong survival, while also inducing immunogenic cell death and enhancing antitumor immunity. Importantly, this process can occur independently of high SLC7A11 expression, thus providing a novel therapeutic target and strategy for glioblastoma distinct from apoptosis and ferroptosis [85].

By contrast, other studies, including transcriptome based risk models and analyses involving sperm-associated antigen 4 (SPAG4) [160], structural maintenance of chromosomes protein 4 (SMC4) [161], immune infiltration, and tumor microbiota [162], lack direct mechanistic evidence such as cystine dependent disulfide accumulation, actin cytoskeletal collapse, and rescue by reducing agents. At present, these findings remain at the level of correlation and their conclusions should therefore be interpreted with caution.

#### 7.1.7 Bone and soft tissue tumors

For osteosarcoma and related mesenchymal tumors, currently available data are still composed mainly of Tier 3 evidence. In osteosarcoma, both LINC01137 [124] and PABPC3 [125] are associated with proliferation, migration, invasion, epithelial-mesenchymal transition (EMT), and immune microenvironment remodeling, and both derive from disulfidptosis related modeling frameworks. However, neither has direct evidence for the core features of disulfidptosis, namely SLC7A11 and cystine dependence, NADPH collapse, abnormal intracellular disulfide accumulation, and destruction of the actin cytoskeleton [124,125]. Therefore, these factors should be interpreted more appropriately as risk model genes associated with disulfidptosis related states rather than as established mechanistic effector molecules. Thus, in bone and soft tissue sarcomas, this field remains at an early stage, and the development of prognostic modeling is currently outpacing mechanistic validation.

#### 7.1.8 Tumor immunity

**Fig. 4:**
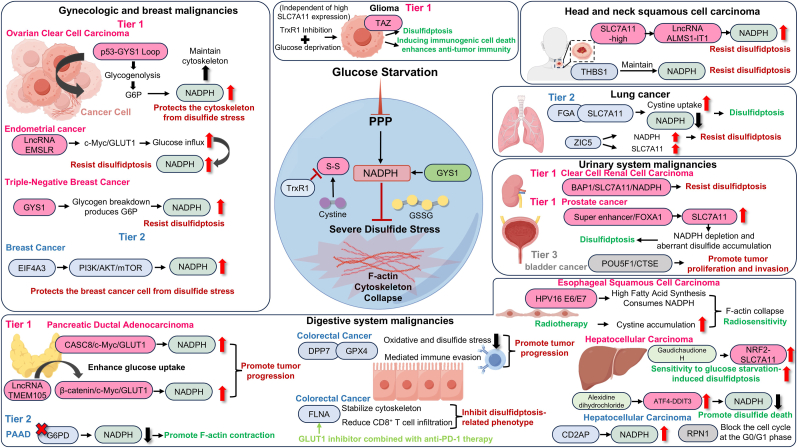
Tumor associated molecular regulators linked to disulfidptosis related phenotypes across cancer types.

Disulfidptosis does not act solely by killing tumor cells. It may also damage immune effector cells within the tumor microenvironment [112]. The STAT3/LDHB/G6PD/PPP axis is a core pathway regulating disulfidptosis of CD8^+^ T cells in the tumor microenvironment. In this setting, disulfidptosis acts as a key mechanism driving immunosuppression, directly causing exhaustion of tumor infiltrating CD8^+^ T cells and weakening antitumor immune responses [112]. By contrast, deletion of LDHB or inhibition of STAT3 can block disulfidptosis and exhaustion of CD8^+^ T cells, restore T cell cytotoxic function, and suppress tumor growth. This is one of the most important findings in nonmalignant cells within the field because it reveals a translational paradox: without adequate metabolic selectivity, therapeutic strategies intended to induce disulfidptosis in tumor cells may also compromise antitumor immunity [70]. Fig. 4 summarizes major tumor associated regulators of disulfidptosis across cancer types, highlighting whether they promote or restrain disulfidptotic outcomes and the strength of current supporting evidence (Fig. 4).

This figure summarizes tumor specific molecular factors associated with disulfidptosis sensitivity or resistance in different malignancies. In gynecologic and breast cancers, the p53/GYS1 loop, GYS1, EIF4A3, and the lncRNA EMSLR increase glycolysis, glucose uptake, or NADPH production, thereby protecting the cytoskeleton from disulfide stress and reducing disulfidptosis susceptibility. In pancreatic ductal adenocarcinoma, CASC8, TMEM105, c-Myc, and G6PD-related regulation reshape glucose metabolism and NADPH availability, thereby influencing disulfidptosis-associated vulnerability. In glioma, TAZ promotes glucose starvation-induced disulfidptosis and immunogenic cell death. In head and neck squamous cell carcinoma, ALMS1-IT1 and THBS1 maintain NADPH homeostasis and are associated with resistance to disulfidptosis. In lung cancer, FGA together with SLC7A11 promotes cystine uptake and favors disulfidptosis, whereas ZIC5 increases NADPH and SLC7A11 expression and opposes disulfidptosis. In urinary system malignancies, BAP1 represses SLC7A11 expression in clear cell renal cell carcinoma reduces cystine influx, preserves NADPH balance under glucose limitation, and thereby suppresses disulfidptosis; super-enhancer/FOXA1-driven SLC7A11 expression in prostate cancer increases vulnerability to glucose deprivation-induced disulfidptosis, thereby facilitating disulfidptosis; and POU5F1 and CTSE in bladder cancer are associated with tumor-promoting phenotypes. In digestive system malignancies, DPP7 and GPX4 in colorectal cancer are linked to tumor progression under oxidative and disulfide stress, while FLNA stabilizes the cytoskeleton and correlates with inhibition of disulfidptosis-related phenotypes. In esophageal squamous cell carcinoma, HPV16 E6/E7 promotes fatty acid synthesis, NADPH consumption, cystine accumulation, and F-actin collapse, thereby enhancing radiosensitivity in a disulfidptosis-related context. In hepatocellular carcinoma, Gaudichaudione H increases sensitivity to glucose starvation-induced disulfidptosis through NRF2/SLC7A11-associated regulation, alexidine dihydrochloride promotes disulfide stress through the ATF4/DDIT3/NADPH axis, CD2AP increases NADPH levels, and RPN1 is linked to G0/G1 cell cycle arrest. Red background boxes indicate Tier 1 evidence, blue background boxes indicate Tier 2 evidence, and gray background boxes indicate Tier 3 evidence. Red font denotes tumor-promoting roles, whereas green font denotes tumor-suppressive roles. Overall, tumor-specific metabolic and transcriptional programs may either facilitate or restrain disulfidptosis-related outcomes depending on context.

### 7.2 Beyond cancer: emerging relevance of disulfidptosis in non-neoplastic diseases

Outside oncology, disulfidptosis is increasingly invoked as a framework for understanding tissue injury under conditions of metabolic collapse and redox disequilibrium. Yet, compared with tumors, the evidence is substantially more heterogeneous and often less definitive. Non-neoplastic diseases should therefore be discussed not as a single validated domain of disulfidptosis, but as a spectrum of contexts in which disulfide-stress-driven structural injury may be mechanistically relevant to varying degrees.

#### 7.2.1 Current evidence across ischemic, inflammatory, degenerative, and toxic injury contexts

The most persuasive non-neoplastic scenario is ischemia-reperfusion and acute bioenergetic failure [[163], [164], [165]]. Stroke and related ischemic injuries recreate several prerequisites for disulfidptosis, including abrupt limitation of glucose utilization, severe NADPH depletion, and oxidative burst upon reperfusion. Within this context, the USP15 and SETD1B axis described in cerebral ischemia offers one of the more mechanistically developed examples, linking epigenetic remodeling to increased expression of actin-regulatory factors such as NCKAP1L and WAVE2 and thereby amplifying structural susceptibility under disulfide stress [86]. Even so, these data are best viewed as strong support for a disulfidptosis-like mechanism unless the full criteria for bona fide structural execution and pathway exclusion are demonstrated.

A second emerging domain is neuronal and neurodevelopmental injury, where bioenergetic fragility and actin dependence make neural cells conceptually susceptible to disulfide-stress-mediated collapse. In models of Parkinsonian toxin exposure [[166], [167], [168]], epilepsy-related excitotoxic injury [116,169], and autism-associated cortical metabolic dysregulation [96], studies have linked altered glucose transport, SLC7A11 upregulation, NADPH insufficiency, and actin destabilization to cell death or dysfunction. The rotenone-induced pathways [167], seizure-associated Rab11a and GLUT3 trafficking failure [116], and cortical glucose transport defects in autism models all align with the broader logic of disulfidptosis. However, because neuronal injury frequently involves overlapping ferroptotic, apoptotic, necrotic, and mitochondrial pathways, most of these contexts currently support compatibility with disulfidptosis rather than definitive assignment of it as the dominant death mechanism.

A third domain comprises inflammatory and barrier disorders, in which disulfide stress may contribute to structural failure of epithelial tissues. Ulcerative colitis and related intestinal inflammatory states are notable because epithelial cells simultaneously experience inflammatory oxidative burden, altered nutrient availability, and disrupted redox homeostasis [123,[170], [171], [172], [173], [174]]. The identification of solute carrier family 3 member 2 (SLC3A2)-centered disulfidptosis-related programs in inflamed intestinal mucosa suggests that epithelial barrier damage may involve a disulfide-stress component, particularly when cytoskeletal proteins become destabilized [123]. SLC3A2, also known as CD98 heavy chain (CD98hc or 4F2hc), is a transmembrane glycoprotein that functions as the heavy-chain subunit of several heteromeric amino acid transporters, including the xCT system, and also participates in integrin-associated signaling [[175], [176], [177]]. Similarly, psoriasis illustrates how disulfidptosis may help explain pathology arising from intratissue metabolic competition: GLUT1-high basal keratinocytes effectively deprive suprabasal cells of glucose, while persistent SLC7A11 expression in the latter creates conditions favorable for disulfide accumulation and impaired differentiation [[178], [179], [180]]. These examples are conceptually strong because they connect tissue architecture to metabolic asymmetry, but they still require careful separation between bona fide death execution and broader stress-associated remodeling.

A fourth domain includes metabolic, toxicological, and multiorgan injury states, where disulfidptosis may function as one component of a larger injury network. Studies in metabolic dysfunction-associated steatotic liver disease (MASLD)/non-alcoholic fatty liver disease (NAFLD) [181,182], acute kidney injury [183], osteoporosis [184], spinal cord injury [[185], [186], [187]], and pollutant-induced thyroid injury [188] all suggest that combined disruption of glucose handling, sulfur metabolism, redox buffering, and cytoskeletal integrity may produce disulfidptosis-like damage. Particularly informative are settings in which a direct perturbation increases cystine influx while simultaneously constraining reducing capacity, as reported for environmental toxicant exposure in thyroid cells or TrxR1 inhibition during osteoclast differentiation [65]. Nevertheless, many of these conditions are currently supported by transcriptomic inference, pathway correlation, or partial mechanistic perturbation rather than full structural proof of disulfidptosis. Their value, therefore, lies less in confirming a universal death mechanism than in expanding the range of biological situations in which disulfide stress may become pathologically consequential.

#### 7.2.2 Future research roadmap for validating disulfidptosis in non-neoplastic diseases

For non-neoplastic diseases, the immediate priority is not to expand the list of putative associations, but to determine whether disulfidptosis truly operates under disease-relevant conditions. A staged validation strategy should begin by mapping baseline susceptibility in relevant primary cells, organoids, ex vivo tissues, or disease models, with attention to SLC7A11-dependent cystine uptake, NADPH/GSH reducing capacity, cystine accumulation, disulfide stress, and actin cytoskeletal vulnerability [1,189]. The next step is to test whether physiologically relevant stressors, such as ischemia–reperfusion, hypoxia, inflammatory stimulation, oxidative or metabolic stress, or pathological glucose limitation, can reproduce the defining features of disulfidptosis within the same vulnerable cell population, rather than relying solely on extreme nutrient deprivation. Pathway specificity should then be established through perturbation, temporal analysis, and mechanism-relevant rescue experiments, including modulation of SLC7A11 or cystine availability, restoration of NADPH-dependent reducing capacity, and prevention or reversal of actin cytoskeletal disulfide damage, while excluding major competing death programs such as apoptosis, ferroptosis, necroptosis, pyroptosis, and other context-relevant modalities [1]. Where feasible, extension to in vivo models will be essential to determine whether disulfidptosis is not only detectable, but also functionally important and therapeutically tractable in non-neoplastic disease [190]. Such a roadmap would help distinguish true disease-operating disulfidptosis from redox-associated correlations and provide a more rigorous basis for biomarker development and therapeutic intervention.

### 7.3 Tissue specificity is shaped by metabolic architecture rather than organ identity alone

One of the clearest lessons from the current literature is that disulfidptotic susceptibility is not dictated by organ identity per se, but by a recurring combination of metabolic load, reductive capacity, and structural dependence that can emerge across very different tissues. Evidence from pancreatic, head and neck, colorectal, liver, ovarian, prostate, neural, bone, and immune cell models indicates that a common vulnerability arises when high cystine influx is coupled to insufficient NADPH buffering and an actin network whose collapse is incompatible with survival. Tissue specificity in this context is therefore better understood as a form of metabolic-structural organization rather than a fixed property of tissue origin.

A central determinant of this architecture is the extent to which a given cell state depends on SLC7A11-driven cystine import. Across multiple tumor types, high SLC7A11 expression creates a latent dependency on continuous disulfide reduction, which becomes hazardous under conditions of glucose limitation or impaired reductive metabolism. This principle is most clearly illustrated in PDAC, HNSCC, and prostate cancer [55,70,159], where elevated cystine uptake is closely linked to disulfidptotic sensitivity once glucose utilization can no longer sustain redox homeostasis. A similar pattern is observed outside epithelial malignancies: in pre-osteoclasts, increased cystine uptake also lowers the threshold for disulfide stress when thiol-reducing systems are compromised [65]. Conversely, in ccRCC, repression of SLC7A11 is associated with reduced susceptibility, underscoring that the magnitude of cystine flux is a primary determinant of whether a tissue enters a disulfidptosis-permissive state [50,191].

A second major variable is the capacity to maintain glucose uptake and NADPH regeneration, particularly through GLUT1-dependent glucose supply and the PPP. In several cancer models, including pancreatic cancer, head and neck cancer, and endometrial cancer, resistance to disulfidptosis is closely associated with preserved glucose influx, enhanced PPP activity, and sustained NADPH availability [70,[76], [77], [78]]. In epileptic neurons, impaired membrane trafficking reduces surface delivery of GLUT3, thereby restricting glucose uptake, depleting NADPH, and promoting disulfide stress [116]. In tumor-infiltrating CD8^+^ T cells, suppression of G6PD, the rate-limiting enzyme of the oxidative PPP, similarly compromises NADPH production [112]. In esophageal squamous cell carcinoma after radiotherapy, excessive NADPH consumption driven by metabolic reprogramming further aggravates cystine burden and favors disulfide accumulation [102]. These findings suggest that tissues differ less in whether they require reducing power than in how effectively they can preserve glucose-derived NADPH under stress.

Metabolic buffering can also be shaped by substrate choice rather than glucose uptake alone. In ovarian clear cell carcinoma, glycogen metabolism becomes a critical reserve that supports PPP flux and replenishes NADPH under cisplatin stress, thereby preserving cytoskeletal integrity and limiting disulfidptotic injury [108]. In hepatocellular carcinoma and lung cancer, related protection appears to involve broader metabolic circuits that maintain redox balance through mitochondrial and biosynthetic adaptation [83,111]. These observations extend the concept of tissue specificity beyond a simple glucose dependence model and indicate that what ultimately matters is whether a cell can mobilize sufficient reducing equivalents to cope with persistent cystine-derived disulfide stress.

Beyond these metabolic dynamics, the structural architecture of the cell is equally critical. Disulfidptosis is distinguished by aberrant disulfide bonding in cytoskeletal proteins, collapse of F-actin, and detachment of the actin cytoskeleton from the plasma membrane. This helps explain why susceptibility repeatedly appears in systems that rely heavily on dynamic cytoskeletal remodeling, including colorectal cancer, neurons, and bone-resorbing cells [65,86,117,146]. These settings suggest that tissue selectivity depends not only on how much disulfide stress accumulates, but also on how severely a given cell type is affected once actin integrity is lost.

Microenvironmental stress further determines when these latent vulnerabilities become phenotypically evident. Glucose deprivation, radiotherapy, ischemia, inflammatory stress, and therapy-induced metabolic competition can all shift a cell from compensated redox adaptation to overt disulfidptotic collapse. This is evident in esophageal squamous cell carcinoma, where radiotherapy exacerbates NADPH stress [102]; in ovarian clear cell carcinoma, where cisplatin challenges reductive homeostasis [108]; and in neurological disorders such as epilepsy and ischemic stroke [192], where interrupted nutrient supply converges on the same endpoint of disulfide overload and cytoskeletal failure. In immune cells, the metabolically restrictive tumor microenvironment likewise creates conditions in which insufficient G6PD-dependent NADPH generation may push CD8^+^ T cells toward exhaustion-associated disulfidptotic damage [112]. Tissue specificity is therefore inseparable from context: an otherwise tolerable metabolic configuration may become lethal when nutrient access, thiol buffering, or redox support is constrained by the local environment.

Taken together, current evidence supports a more precise interpretation of tissue specificity in disulfidptosis. What matters most is not whether a cell originates from the pancreas, ovary, liver, prostate, brain, bone, or intestinal mucosa, but whether it adopts a state characterized by high cystine flux, inadequate or overcommitted NADPH regeneration, dependence on GLUT1-or G6PD-supported metabolic buffering, and strong reliance on actin cytoskeletal integrity. This framework explains why mechanistically diverse diseases converge on a common endpoint and why markers such as SLC7A11, GLUT1, and G6PD may be more informative than organ classification alone when predicting vulnerability [1]. At the same time, the strength of evidence remains uneven across disease categories. While several cancer and neural models now provide strong mechanistic support, observations in other settings remain more preliminary because they still lack definitive demonstration of disulfide accumulation, actin collapse, rescue by reducing agents, or rigorous exclusion of competing death pathways. For this reason, tissue specificity in disulfidptosis is best viewed, at present, not as an organ-based taxonomy, but as a testable model of metabolic-structural vulnerability.

## 8 Evidence stratification of disulfidptosis-targeted interventions

**Fig. 5:**
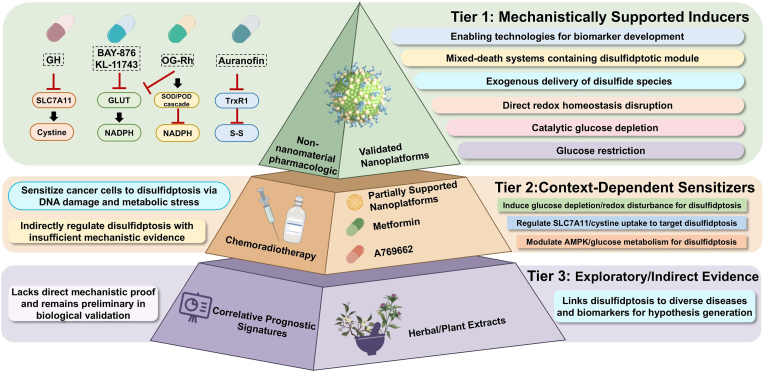
Evidence based stratification of translational interventions targeting disulfidptosis.

To better define the translational relevance of disulfidptosis, current evidence can be organized into a level-of-evidence framework rather than a simple catalogue of candidate interventions. Such a structure helps distinguish mechanistically validated preclinical strategies from context-dependent or exploratory observations, and aligns translational expectations with the strength of pathway attribution (Fig. 5, Table 3).

**Table 3:** Engineered and pharmacologic interventions used to induce or interrogate disulfidptosis.

Category	Ref.	Intervention/system	Mechanistic entry point	Key evidence supporting disulfidptosis	Causal validation/rescue	Competing death pathways assessed	Evidence tier for disulfidptotic module	Most cautious interpretation
Canonical metabolic trigger	[1,193]	BAY-876 or KL-11743-based glucose uptake inhibition	Direct restriction of glucose uptake and reducing power	Disulfidptosis-compatible metabolic context and structural injury in SLC7A11-high cells	Pathway-relevant rescue reported	Alternative pathways partly assessed	Tier 1	Strong translational extension of the canonical model
Small-molecule inducer	[85]	TrxR1 inhibitors (auranofin, TRi-1)	Impaired thiol-reducing capacity under glucose starvation	Cystine accumulation; actin collapse; disulfidptosis-like death in a permissive metabolic context	DTT rescue	Apoptosis and ferroptosis reasonably excluded	Tier 1	Strong evidence that impaired thiol reduction can trigger disulfidptosis
Small-molecule inducer	[147]	Gaudichaudione H	NRF2/SLC7A11-linked enhancement of disulfidptotic susceptibility	NADPH depletion; actin injury; disulfidptosis-related death	Rescue evidence reported	Competing pathways partly addressed	Tier 1	Mechanistically persuasive sensitizer of disulfidptosis
Small-molecule inducer	[194]	OG-Rh	Metabolic sabotage and redox catalysis	NADPH depletion; structural injury; disulfidptosis-associated death	Reductive rescue reported	Apoptotic crosstalk present	Tier 1	Supports a disulfidptotic module within mixed death
Small-molecule inducer	[148]	Alexidine dihydrochloride	ATF4-DDIT3-linked sensitization to cystine-related metabolic stress	Mitochondrial stress, ATF4/DDIT3 activation, impaired amino acid/GSH metabolism, reduced NADPH, disulfide stress, and cytoskeletal collapse	ATF4 or DDIT3 knockdown reverses the sensitizing effect	Alternative pathways not fully excluded	Tier 1	Credible sensitizer, but mechanistic closure is less complete than canonical models
Small-molecule or molecular intervention	[159]	THBS1-targeted intervention	Increased disulfide accumulation during glucose starvation and modulation of NADPH balance	Disulfide accumulation, F-actin contraction, and SLC7A11-related phenotypes reported	TCEP rescue reported	Competing pathways less comprehensively resolved	Tier 2	Supportive of a disulfidptotic component, but not fully pathway-definitive
Engineered dual-input system	[195]	CYBC nanoparticles	Combined GLUT1 inhibition and cystine delivery	Canonical metabolic context; cystine burden; actin disruption; in vivo efficacy	TCEP, DTT, or β-mercaptoethanol rescue	Ferroptosis, apoptosis, and ROS-driven alternatives assessed	Tier 1	Among the strongest engineered demonstrations of disulfidptosis
Engineered dual-input system	[196]	GOx/CuSAE hybrid nanozyme	Glucose depletion plus consumption of reducing power	Disulfide accumulation, NADPH depletion, and actin contraction	Rescue by reducing interventions	Cuproptosis also engaged	Tier 1	Strong evidence for disulfidptosis in a mixed disulfidptosis-cuproptosis system
Engineered dual-input system	[197]	HGV deformable nanocapsules	Amplified starvation stress with metabolic deception	Metabolic disruption; disulfide stress; cytoskeletal injury	Rescue by TCEP or nutrient restoration	Competing pathways partly examined	Tier 1	Strong engineered support for disulfidptosis-like execution
Engineered dual-input system	[198]	GOx@HPB nanomotor	GOx-mediated glucose depletion and redox disruption	Cystine accumulation; NADPH depletion; cytoskeletal injury	DTT rescue	Pathway exclusion performed to a meaningful extent	Tier 1	Strong support for catalytic glucose depletion-induced disulfidptosis
Engineered dual-input system	[199]	ITG@PCM nanoreactor	Reprogramming of the cystine-GSH axis with glucose, NADPH, and GSH depletion	Cystine-dependent disulfide stress and cytoskeletal destruction	Rescue evidence reported	Pyroptosis component present	Tier 1	Mixed-death system containing a validated disulfidptotic module
Engineered dual-input system	[200]	Cys-hMnO2@GOx@EM-CD24	CD24-targeted cystine delivery plus GOx-mediated glucose depletion	Combined cystine loading and glucose depletion required for full disulfidptotic lethality in neuroblastoma	Evidence supports dependence on combined cystine and glucose-depletion inputs	Other stress mechanisms possible but disulfidptotic module strongly supported	Tier 1	Strong dual-input nanosystem supporting disulfidptosis induction
Direct redox/disulfide sabotage	[201]	NI@PSL nanoparticles	NADPH consumption plus lipoate-derived disulfide exchange stress	Cytoskeletal disulfide crosslinking and disulfide stress-associated death	TCEP rescue; diamide sensitization	Nonexclusive oxidative contributions possible	Tier 1	Strong evidence for direct disulfide-stress amplification
Mixed-death system with validated disulfidptotic component	[202]	RuSSRu ruthenium complex	SLC7A11-related cystine/disulfide stress under photoactivation	Actin collapse and disulfidptosis-related injury	Rescue evidence reported	Apoptotic component present	Tier 1	Mechanistically supportive, but mixed with apoptosis
Mixed-death system with validated disulfidptotic component	[203]	Tumor-directed copper bioorthogonal activation system	Spatially controlled copper-triggered disulfide stress	Disulfidptosis-compatible redox and structural injury phenotype	Rescue evidence reported	Cuproptosis overlap possible	Tier 1	Supports inducible disulfidptosis with possible copper-related overlap
Mixed-death system with validated disulfidptotic component	[204]	GOx-IA@HMON@IO or related platform	Glucose depletion with concurrent ferroptotic pressure	Disulfidptosis-relevant metabolic stress and actin injury	Reductive rescue for the disulfidptotic component	Ferroptosis explicitly co-engaged	Tier 1	Combinatorial ferroptosis-disulfidptosis therapy rather than pure disulfidptosis
Mixed-death system with validated disulfidptotic component	[205]	FCSP@876	GLUT inhibition plus ferroptotic reinforcement	Disulfide stress and actin damage in SLC7A11-vulnerable cells	Reductive rescue reported	Ferroptosis coexists	Tier 1	Validates a disulfidptotic module within dual death design
Mixed-death system with validated disulfidptotic component	[206]	4T1@MFCB biomimetic nanoagonist	Coinhibition of glucose metabolism and the SLC7A11/GSH/GPX4 axis	Disulfide stress; actin disruption; cystine-dependent context	Rescue experiments support mechanism attribution	Ferroptotic signaling also present	Tier 1	Mixed ferroptosis-disulfidptosis system
Mixed-death system with validated disulfidptotic component	[207]	CCD@RF nanoparticles	Suppression of glucose metabolism with copper-driven stress	Rescuable disulfide stress and actin damage; in vivo support	Reductive rescue	Cuproptosis and immunogenic death features present	Tier 1	Strong disulfidptotic component within a multimodal response
Mixed-death system with validated disulfidptotic component	[208]	CuSS@876-PEG or copper-MOF strategy	SLC7A11-dependent disulfide stress with glucose restriction	Actin cytoskeletal disorder and disulfide stress in a permissive context	Rescue evidence reported	Cuproptosis component present	Tier 1	Combination-death system with credible disulfidptosis
Mixed-death system with validated disulfidptotic component	[209]	CST NPs/related copper-driven dual-death system	Redox imbalance and cystine-related structural injury	F-actin collapse in a cystine uptake-dependent setting	Redox rescue and pathway testing	Pyroptosis or copper-associated mixed death present	Tier 1	Disulfidptotic module is supported, but not exclusive
Mixed-death system with validated disulfidptotic component	[210]	CGBH NNs	Glucose consumption with SLC7A11-dependent disulfide stress	Cytoskeletal injury and disulfidptosis-relevant readouts	Reductive rescue	Pyroptosis component present	Tier 1	Mixed-death nanoplatform with a validated disulfidptotic arm
Mixed-death system with validated disulfidptotic component	[211]	Programmable prodrug nanomodulator	Tumor redox disruption to amplify disulfidptosis and pyroptosis	Disulfidptosis-compatible structural and redox features	Rescue evidence reported	Pyroptosis intentionally co-induced	Tier 1	Pyroptosis plus a validated disulfidptotic module
Mixed-death system with partial mechanistic closure	[212]	M@GOx/Fe-HMON hybrid nanoenzyme	Glucose dyshomeostasis with ferroptotic reinforcement	Disulfidptosis-compatible phenotype present	Canonical rescue chain incomplete	Ferroptosis strongly involved	Tier 2	Suggestive mixed ferroptosis-disulfidptosis system
Mixed-death system with partial mechanistic closure	[213]	CuAp@GOx nanoshuttle	Glucose dyshomeostasis with cuproptosis, ferroptosis, and disulfidptosis features	Mixed metabolic death phenotype partly supportive of disulfidptosis	Direct validation of terminal structural hallmarks incomplete	Cuproptosis and ferroptosis coexist	Tier 2	Disulfidptosis-compatible rather than disulfidptosis-defining
Context-dependent candidate	[214]	MTFGM nanoreactor	Glucose/GSH depletion with cystine accumulation and redox imbalance	Strong upstream stress phenotype, but terminal actin-disulfide execution not fully validated	No full canonical rescue chain	Mixed oxidative death remains possible	Tier 2	Mechanistically suggestive but not definitive
Context-dependent candidate	[215]	SPCP/CCP@Bay	Selective vulnerability linked to cystine metabolism and GLUT inhibition	Supports cystine-dependent vulnerability	Definitive actin-collapse evidence limited	Alternative death programs incompletely resolved	Tier 2	Supports pathway involvement without full mechanistic closure
Context-dependent candidate	[216]	HA/PLL crosslinked hydrogel	Perturbation of glutamate/cystine-related metabolism with cytoskeletal collapse	Cystine accumulation and actin damage reported	Specific biochemical rescue lacking	Broader stress-related injury not excluded	Tier 2	Disulfidptosis-like phenotype with incomplete specificity
Context-dependent candidate	[217]	SC@Lip nanoliposomes	Sonodynamic oxidative stress with SLC7A11-associated vulnerability	Disulfidptosis-like phenotype proposed	Terminal execution phase insufficiently confirmed	Lipid peroxidation and ROS contributions likely	Tier 2	Better described as disulfidptosis-compatible
Context-dependent candidate	[218]	Cell-cycle arrest agents or PARP inhibitors	Increased susceptibility to glucose starvation-associated death	Supports metabolic sensitization to disulfidptosis	Full structural confirmation limited	Alternative stress-death pathways remain plausible	Tier 2	Better viewed as sensitizers than direct inducers
Context-dependent candidate	[192]	Metformin in ischemic stroke models	Modulation of metabolic stress and neuronal protection	Disulfidptosis-related markers affected	Structural execution and rescue framework incomplete	Multiple neuroprotective pathways likely involved	Tier 2	Biologically relevant but not definitive for disulfidptosis modulation
Context-dependent candidate	[219]	A769662-associated metabolic models	AMPK activation with altered SLC7A11-dependent redox handling	Compatible upstream metabolic perturbation	Full structural execution not consistently shown	Competing pathways incompletely assessed	Tier 2	Supportive of the model but less definitive than canonical studies
Indirect or exploratory evidence	[171]	Modified Gegen Qinlian decoction	Modulation of disulfidptosis-related gene networks	Mainly transcriptomic or correlative evidence	No canonical rescue or structural validation	Alternative mechanisms highly plausible	Tier 3	Hypothesis-generating only
Indirect or exploratory evidence	[220]	3′-Methoxydaidzein	Association with disulfidptosis-related molecular clusters	Indirect network-level support only	No direct mechanistic validation	Competing explanations not excluded	Tier 3	Exploratory pharmacologic association
Indirect or exploratory evidence	[184]	Kaempferol	Association with a disulfidptosis-related target	No direct biochemical or structural evidence	No rescue-based validation	Alternative mechanisms unresolved	Tier 3	Insufficient for mechanistic assignment
Indirect or exploratory evidence	[221,222]	Cardiomyocyte metabolic-stress models	Multi-omics association with disulfidptosis-like signatures	Indirect signature-level support only	No canonical mechanistic testing	Highly nonspecific stress context	Tier 3	Useful for hypothesis generation, not pathway confirmation
Enabling technology	[223]	PPCA/Pt/CFNE single-cell cysteine nanosensor	Functional measurement of cysteine/disulfide metabolic state	Links cysteine flux to disulfidptosis susceptibility in SLC7A11-high cells	Supported by cystine withdrawal or 2-DG-type perturbation logic	Not a death-induction study	Tier 1	Enabling tool for stratification rather than a direct inducer

**Interpretive note:** Some Tier 1 interventions also co-engage ferroptosis, cuproptosis, pyroptosis, or apoptosis. Their assignment reflects the strength of evidence for the disulfidptosis component rather than pathway exclusivity. Tier assignment was interpretive and narrative rather than statistically weighted; it was based on mechanistic convergence across reported experiments.

This figure summarizes translational strategies related to disulfidptosis according to the strength and directness of available evidence. Tier 1 includes mechanistically supported inducers. Representative interventions shown in this tier include Gaudichaudione H (GH), BAY-876, KL-11743, OG-Rh, and auranofin, which act through SLC7A11 and cystine metabolism, GLUT and NADPH-related glucose utilization, the SOD/POD cascade and NADPH regulation, or TrxR1 and disulfide-related redox control. This tier also highlights enabling technologies for biomarker development, mixed death systems containing a disulfidptotic module, exogenous delivery of disulfide species, direct disruption of redox homeostasis, catalytic glucose depletion, glucose restriction, and nanoplatform-based or non-nanomaterial pharmacologic approaches. Tier 2 includes context-dependent sensitizers, including chemoradiotherapy, nanoplatforms, metformin, and A769662, which may sensitize cancer cells to disulfidptosis through DNA damage, metabolic stress, glucose depletion, redox disturbance, regulation of SLC7A11 and cystine uptake, or modulation of AMPK and glucose metabolism, but with insufficient direct mechanistic support in some settings. Tier 3 represents exploratory or indirect evidence, including correlative prognostic signatures and herbal or plant extracts, which currently lack direct mechanistic proof and mainly support hypothesis generation linking disulfidptosis to diverse diseases and biomarkers. Overall, the figure distinguishes interventions with direct mechanistic support from those that remain context-dependent or primarily exploratory.

### 8.1 Mechanistically supported preclinical rationale

At present, most Tier 1 evidence comes from preclinical oncology studies, particularly in metabolically vulnerable, SLC7A11-high tumors. For clarity, these interventions can be organized according to their dominant entry point into the pathway: canonical metabolic triggers such as glucose restriction or GLUT inhibition; engineered nanoplatforms that recreate disulfidptosis-permissive conditions in vivo; non-nanomaterial pharmacologic sensitizers that perturb redox buffering or cystine-disulfide handling; mixed-death systems in which a validated disulfidptotic module coexists with other regulated death programs; and enabling technologies that facilitate mechanistic resolution or biomarker-guided stratification.

#### 8.1.1 Canonical metabolic triggers: glucose limitation and GLUT inhibition

Studies using BAY-876, KL-11743, and related approaches showed that when cystine uptake remains high but glucose utilization is restricted, tumor cells become selectively vulnerable to disulfidptosis [1,193]. In these settings, impaired glucose flux limits PPP-dependent NADPH regeneration, prevents efficient reduction of accumulated intracellular disulfides, and culminates in F-actin contraction and cytoskeletal collapse. This remains the clearest translational embodiment of the core biological logic of disulfidptosis.

Related work further suggests that redox adaptation may generate a therapeutically exploitable state of metabolic death plasticity. Tumors that acquire ferroptosis resistance through persistent activation of the SLC7A11/GSH/GPX4 axis may become increasingly dependent on NADPH and thus become conditionally susceptible to disulfidptosis under abrupt glucose restriction or blockade of compensatory reducing systems such as TrxR1 [63,85]. This concept is attractive because it reframes disulfidptosis not simply as an isolated death pathway, but as a context-dependent metabolic liability that may emerge after adaptation to other redox-active therapies. Even so, it should still be presented as a mechanistically plausible preclinical model rather than a clinically validated therapeutic strategy.

#### 8.1.2 Nanoplatforms that recapitulate canonical disulfidptosis conditions

Although these systems vary considerably in composition, delivery mode, and auxiliary functions, they can be interpreted within a shared analytical framework encompassing six dimensions: the route of metabolic entry into disulfidptosis, the extent to which canonical mechanistic criteria are fulfilled, whether additional death programs co-occur, the availability of in vivo antitumor evidence, the presence of rescue experiments or pathway-specific validation, and the principal translational limitations. This framework helps distinguish systems that predominantly model bona fide disulfidptosis from those in which disulfidptosis is embedded within a more complex mixed-death phenotype.

Among the strongest examples are platforms that simultaneously impose glucose restriction and increase cystine burden. Cancer cell membrane-camouflaged copper-cystine nanoparticles coloaded with BAY-876 (CYBC NPs) exemplify this strategy by combining GLUT1 inhibition with direct cystine delivery [195]. In this system, glucose restriction led to NADPH depletion, intracellular cystine accumulation, and abnormal disulfide-mediated disruption of the actin cytoskeleton, while reducing agents such as TCEP, DTT, and β-mercaptoethanol robustly rescued cell viability and cytoskeletal integrity [195]. Importantly, ferroptosis inhibitors, apoptosis inhibitors, and ROS scavengers failed to reverse the lethal phenotype, strengthening the attribution to canonical disulfidptosis rather than to nonspecific oxidative collapse or a mixed oxidative death program [195]. In vivo, CYBC NPs markedly inhibited tumor growth, suppressed lung metastasis, prolonged survival, and showed acceptable short-term biosafety in 4T1 models. However, important translational constraints remain, including manufacturing complexity, the reproducibility of homologous membrane coating, and limited evaluation of long-term copper-related toxicity.

A related but mechanistically distinct subset uses catalytic glucose depletion rather than transporter inhibition as the dominant trigger. Glucose oxidase-loaded platforms such as GOx@HPB [198], HGV [197], ITG@PCM [199], and Cys-hMnO2@GOx@EM-CD24 [200] exploit GOx-mediated glucose consumption to lower NADPH availability and thereby sensitize tumor cells to disulfide stress. Glucose oxidase (GOx) is a fungal oxidoreductase widely used in nanomedicine because it catalyzes glucose oxidation to gluconic acid and hydrogen peroxide, thereby depleting intratumoral glucose and reinforcing oxidative or metabolic stress [[224], [225], [226]]. In the strongest examples, canonical readouts include NADPH depletion, intracellular cystine or disulfide accumulation, F-actin collapse, and rescue by DTT or TCEP. GOx@HPB [198], for instance, showed DTT-reversible disulfidptotic death together with strong in vivo efficacy in hepatoma models. HGV further intensified nutrient stress through simultaneous Alanine, Serine, Cysteine Transporter 2 (ASCT2) inhibition and showed rescue by TCEP and nutrient replenishment [197]. Cys-hMnO2@GOx@EM-CD24 provided especially persuasive causal support by demonstrating that glucose depletion alone or cystine delivery alone was insufficient, whereas their combination was required to induce full disulfidptotic lethality in neuroblastoma [200].

Another subgroup of Tier 1 nanomedicines amplifies disulfidptosis through direct redox sabotage or exogenous disulfide delivery. Nitroimidazole-grafted poly(sodium lipoate) nanoparticles (NI@PSL) are particularly informative because they induce disulfide stress through two coordinated mechanisms: GSH-responsive degradation releases lipoate-derived species that can promote abnormal disulfide exchange with cytoskeletal proteins, while hypoxia-activated nitroimidazole reduction consumes NADPH and further impairs disulfide reduction capacity [201]. In this model, TCEP rescued cell death in a dose-dependent manner, diamide enhanced toxicity, and nonreducing/reducing immunoblotting directly supported abnormal cytoskeletal disulfide crosslinking. A related logic is observed in GOx/CuSAE, in which GOx-driven glucose deprivation is coupled to further depletion of reducing equivalents [196]. Such systems strongly support a disulfidptotic mechanism, although some also display a mild ferroptotic component, underscoring the need to specify whether disulfidptosis is the dominant driver or one element within a broader composite death response.

Nevertheless, major translational barriers persist, including enzyme instability, multistep fabrication, dependence on enhanced permeability and retention effects, and uncertain long-term biodistribution and clearance.

#### 8.1.3 Non-nanomaterial pharmacologic sensitizers and pathway modulators

Tier 1 evidence is not confined to nanotherapeutic systems. In parallel with these engineered platforms, a smaller but important body of work indicates that disulfidptotic signaling may also be pharmacologically induced or reinforced by non-nanomaterial interventions acting on specific nodes of redox control, nutrient stress, or cytoskeletal vulnerability.

In glioblastoma, TrxR1 inhibitors such as auranofin and TRi-1 have been reported to aggravate disulfide stress and promote disulfidptosis-like death under glucose-starved conditions [85]. These findings support the idea that inhibition of compensatory thiol-reducing capacity can function as an upstream sensitizing mechanism, especially when glucose-derived reducing equivalents are already limited. Mechanistically, this aligns well with the broader Tier 1 framework, in which the inability to reduce accumulated intracellular disulfides is central to actin-cytoskeletal injury and cell death.

In hepatocellular carcinoma, compounds such as Gaudichaudione H (GH), and nitroxyl radical-conjugated Rh(III) complex (OG-Rh) have likewise been associated with disulfide accumulation, cytoskeletal injury, and growth suppression consistent with disulfidptosis-associated mechanisms [147,194]. Similarly, THBS1-targeting strategies in head and neck squamous cell carcinoma have been reported to affect disulfidptosis-associated phenotypes, although the mechanistic depth remains less complete than in canonical Tier 1 studies [159]. Collectively, these studies are important because they extend Tier 1 evidence beyond a purely nanomaterial-centered literature and suggest that disulfidptosis may be pharmacologically inducible through multiple upstream nodes.

At the same time, these non-nanomaterial systems are heterogeneous in biological setting and mechanistic depth. Not all studies provide equally complete documentation of NADPH depletion, intracellular disulfide accumulation, actin-disulfide remodeling, rescue by reducing agents, or exclusion of competing death pathways. Accordingly, for some reports, terms such as “disulfidptosis-like” or “disulfidptosis-associated” may be more appropriate than definitive claims of canonical disulfidptosis. This distinction is important for preserving evidentiary rigor while still recognizing the translational relevance of these pharmacologic observations.

#### 8.1.4 Mixed-death systems containing a validated disulfidptotic module

Several studies have described interventions satisfying canonical disulfidptosis criteria while simultaneously activating other regulated cell death programs. These mixed-mechanism systems remain mechanistically informative and translationally relevant, but causal attribution should be stated with precision. In such cases, the most appropriate interpretation is often not that the intervention induces “pure” disulfidptosis, but rather that it contains a validated disulfidptotic module within a broader combinatorial death network.

For example, GOx-IA@HMON@IO [204], FCSP@876 [205], and 4T1@MFCB [206] combine glucose depletion or GLUT1 inhibition with iron-dependent lipid peroxidation and therefore induce disulfidptosis together with ferroptosis rather than disulfidptosis alone. In these systems, disulfide-reducing agents rescue the disulfidptotic component, whereas ferroptosis inhibitors such as ferrostatin-1 or deferoxamine reverse the ferroptotic component. Likewise, CCD@RF NPs [207], Cadict [227], Ftn@Cu/rhein [203], CuSS@876-PEG [208], and CST NPs [209] combine cystine overload or glucose restriction with copper-triggered mitochondrial proteotoxic stress, thereby functionally linking disulfidptosis with cuproptosis. Other systems, including Pro@FLNC [211], ITG@PCM [199], and CGBH NNs [210], integrate disulfidptosis with pyroptosis, whereas RuSSRu [202] and OG-Rh [194] engage disulfidptotic and apoptotic signaling in parallel.

These studies should therefore be presented as combination-death strategies containing a mechanistically supported disulfidptotic component, rather than as evidence for isolated disulfidptosis. This distinction is not merely semantic. It is essential for defining evidence boundaries, because translational success may ultimately depend not only on whether disulfidptosis is activated, but also on how it interacts with ferroptosis, cuproptosis, pyroptosis, or apoptosis in specific metabolic and therapeutic contexts.

#### 8.1.5 Enabling technologies for biomarker development and stratification

Tier 1 evidence also includes enabling technologies that may facilitate mechanistic resolution and patient stratification rather than directly serving as therapies. One notable example is the single-cell electrochemical nanosensor PPCA/Pt/CFNE [223], which quantitatively tracks intracellular cysteine dynamics and may help predict disulfidptosis susceptibility [1,223]. In SLC7A11-high cells, glucose deprivation or BAY-876 exposure produced characteristic cysteine/disulfide metabolic changes, and sensitivity could be linked functionally to cystine dependence, SLC7A11 status, and rescue by metabolic interventions such as cystine withdrawal [1,15,16,85,196].

Although such tools are not therapeutic agents, they address a major translational bottleneck in the field: the absence of robust biomarkers for identifying tumors that truly satisfy the metabolic prerequisites for disulfidptosis in vivo [223,228,229]. This is particularly important because the strongest preclinical responses have generally been observed in highly selected contexts, especially tumors with high SLC7A11-dependent cystine flux and insufficient flexibility in NADPH regeneration [1,228,229]. Without stratification tools of this type, translation into clinical settings risks becoming empirically driven rather than mechanism-guided.

Taken together, Tier 1 studies support the view that disulfidptosis is most translationally compelling when three conditions are simultaneously met: first, a biologically plausible metabolic context, particularly high SLC7A11-dependent cystine flux [1,228]; second, a defined trigger of reductive failure, most commonly glucose deprivation, GLUT inhibition, or blockade of compensatory thiol-reducing systems [1,85,196]; and third, direct evidence that cell death derives from actin-associated disulfide stress rather than from nonspecific oxidative injury or generalized metabolic collapse [196]. In practice, the strongest Tier 1 studies are those that combine a clearly defined metabolic entry point with evidence of NADPH failure, intracellular disulfide accumulation, structural confirmation of actin-cytoskeletal collapse, and pathway-specific rescue by reducing agents or equivalent interventions [39,195]. By contrast, studies lacking such mechanistic closure, or those dominated by concurrent ferroptotic, cuproptotic, pyroptotic, or apoptotic signaling, should be interpreted more cautiously even if they remain highly relevant to therapeutic design [230].

Overall, the field faces consistent and critical translational barriers: overreliance on preclinical animal models, incomplete pharmacokinetic and long-term toxicity profiling, complex manufacturing of advanced delivery systems, limited investigation into synergies with chemotherapy and targeted therapies, and the outstanding challenge of identifying human tumors that genuinely fulfill the metabolic prerequisites for disulfidptosis in vivo [195,205,215].

### 8.2 Context-dependent candidates with partial mechanistic support

This category includes several drug sensitizers and combination strategies. Agents such as hydroxyurea [218], 5-fluorouracil [218,231,232], nocodazole [218], and poly(ADP-ribose) polymerase (PARP) inhibitors [218] have been reported to increase susceptibility to glucose deprivation- or GLUT inhibition-associated death, suggesting that disulfidptosis may intersect with replication stress, cell-cycle perturbation, and DNA damage response pathways. However, in most such studies, the contribution of disulfidptosis is inferred rather than definitively established. Thus, these interventions are better regarded as context-dependent modulators of disulfidptotic vulnerability rather than validated disulfidptosis-inducing therapies.

Another distinct subcategory of partially validated interventions encompasses metabolic platforms and systems based on biomaterials that are compatible with disulfidptosis but do not yet fully satisfy stringent mechanistic criteria. Representative examples include hydrogels composed of hyaluronic acid (HA) and poly-l-lysine (PLL) [216], sorafenib-chlorin e6 (SC) co-loaded liposomes (SC@Lip) [217], the MTFGM nanoreactor [214], SPCP and CCP polymeric complexes loaded with BAY876 [215], copper apatite (CuAp) structures delivering GOx [213], and hollow mesoporous organosilica nanoparticles (HMON) doped with iron (Fe), loaded with GOx, and camouflaged by cellular membranes [212]. These platforms often induce glucose consumption, oxidative-redox disturbance, or disulfide stress, and some demonstrate tumor growth inhibition consistent with a disulfidptosis-like mechanism. Nevertheless, because multiple death pathways may operate simultaneously, especially ferroptosis, cuproptosis, apoptosis, or general oxidative injury, these studies are more appropriately classified as partial rather than definitive evidence.

Tier 2 also includes interventions in non-cancer disease settings where disulfidptosis has been proposed but remains incompletely resolved. For instance, metformin in ischemic stroke-related neuronal injury [192] enhances mitochondrial complex I function by upregulating Ndufa11 and strengthening its interaction with Ndufs1, while concurrently reducing SLC7A11 expression, lowering the NADP^+^/NADPH ratio, and limiting cystine uptake, changes that collectively mitigate disulfidptosis-associated stress. Similarly, A769662 in glioblastoma-associated metabolic models suggests that modulation of glucose utilization, AMPK signaling, cystine transport, and redox homeostasis can influence disulfidptosis-related phenotypes [219]. Yet these observations are strongly context-dependent and may reflect broader metabolic stress responses rather than selective engagement of the canonical disulfidptosis pathway.

Accordingly, Tier 2 evidence should be interpreted as hypothesis-strengthening but not pathway-confirming. These studies are valuable because they broaden the therapeutic and disease landscape of disulfidptosis, but they also underscore the need for stricter standards of pathway validation.

### 8.3 Indirect, correlative, or exploratory evidence

This level includes disease-association studies and exploratory pharmacologic observations in conditions such as ulcerative colitis [171,220], osteoporosis [184], cardiovascular stress [221,222], and other non-oncologic contexts. Examples include reports involving 3′-methoxydaidzein [220], modified Gegen Qinlian decoction [171], and kaempferol [184], as well as broader analyses linking disulfidptosis-related genes to inflammatory or degenerative phenotypes [122,155,162,233,234]. Such studies are useful for expanding the conceptual scope of disulfidptosis, but in the absence of direct evidence for disulfide-driven actin collapse and pathway-specific rescue, they should be considered preliminary.

Beyond exploratory pharmacological observations, this level of evidence also encompasses correlative biomarkers or risk signatures incorporating genes such as ChaC glutathione specific gamma-glutamylcyclotransferase 1 (CHAC1) [191], cyclin B2 (CCNB2) [235], or other disulfidptosis-related candidates [121,[124], [125], [126],^236^]. While these signatures may carry prognostic or stratification value, they do not by themselves prove pathway activation, dependence, or therapeutic tractability. Biomarker association must therefore be distinguished from mechanistic attribution.

Overall, Tier 3 evidence is best viewed as a source of testable hypotheses. It can guide model selection, biomarker discovery, and disease prioritization, but it should not yet be used as a standalone basis for translational claims.

### 8.4 Clinically tractable modulators of disulfidptosis

From a translational perspective, the most promising disulfidptosis modulators are not necessarily those with the strongest in vitro activity, but those supported by mechanistic credibility, acceptable pharmacokinetic properties, tumor selectivity, and a feasible therapeutic window. Current candidates can therefore be broadly divided into near-term tractable strategies and more exploratory approaches.

Among available agents, inhibitors of thiol-reducing systems, particularly TrxR-targeting drugs such as auranofin, are currently among the most clinically tractable candidates because they combine direct mechanistic relevance with available human safety and pharmacokinetic information [85]. However, their limited tumor selectivity remains a major concern, suggesting that biomarker-guided patient selection and rational combination strategies will likely be required. Agents targeting glucose uptake, glycolytic flux, or glucose-derived NADPH regeneration also have strong mechanistic rationale because they closely mimic the canonical metabolic trigger of disulfidptosis [76]. Nevertheless, their broad physiological roles create substantial challenges in therapeutic selectivity and systemic tolerability.

Alongside targeted experimental agents, several approved and repurposed drugs acting on disulfidptosis-associated metabolic nodes are currently under investigation for their pathway-modulating potential. Importantly, in non-neoplastic diseases, these agents may exert cytoprotective effects by suppressing disulfidptosis, thereby preserving cellular integrity and attenuating pathological tissue injury. Although N-acetylcysteine (NAC) [237], cysteamine [238], and sulfasalazine [239] were not originally developed as specific disulfidptosis modulators, each intersects mechanistically with core components of the pathway. For instance, NAC directly reduces aberrant disulfide bonds via thiol-disulfide exchange and has advanced to Phase II clinical trials for oxidative stress-related conditions; its protective efficacy is partly attributable to the mitigation of disulfidptosis-mediated cytoskeletal collapse [237]. Similarly, cysteamine forms mixed disulfides to deplete free cystine from lysosomal compartments. Already clinically approved for cystinosis, this agent may theoretically restrict the substrate supply required for disulfidptosis, thereby inhibiting the lethal cascade in non-tumor contexts [238]. Sulfasalazine acts as a pharmacological inhibitor of SLC7A11; however, because its primary cytotoxic mechanism stems from ferroptosis induction, its overall impact on disulfidptosis remains highly context-dependent [239]. In non-neoplastic conditions, its cystine-limiting action could theoretically mitigate intracellular disulfide stress, although the concurrent risk of triggering ferroptosis must be carefully weighed.

Collectively, these observations suggest that the near-term clinical translation of disulfidptosis-targeting strategies may rely heavily on repurposing existing therapeutics in combination with biomarker-driven patient stratification, particularly when deploying them as protective interventions against non-malignant disorders. Nonetheless, substantial translational barriers persist, such as suboptimal target selectivity, systemic toxicity risks, and complex mechanistic crosstalk [147,196]. These challenges necessitate rigorous validation in disease-specific models to delineate clear efficacy and safety boundaries.

## 9 Translational opportunities and challenges in disulfidptosis-based therapy

### 9.1 Therapy-oriented combinations: chemotherapy, radiotherapy, and immunotherapy

A therapy-oriented view of disulfidptosis is important because, although clinically actionable modulators may provide entry points for pathway engagement, their near-term translational value is likely to be best realized through rational combination strategies rather than broad standalone use [229,240]. In this context, disulfidptosis is most plausibly exploited as a sensitizing mechanism that enhances the efficacy of established therapies or helps overcome resistance shaped by redox adaptation.

Chemotherapy may create such opportunities by increasing oxidative and metabolic stress and by exposing dependence on NADPH buffering. Thus, in tumor cells characterized by high cystine uptake or limited reductive buffering capacity, certain therapeutic agents that increase ROS burden, disrupt glutathione metabolism, or impose replication-associated metabolic stress may lower the threshold for disulfide stress-associated injury and provide potential opportunities for combination strategies aimed at inducing disulfidptosis [229,241,242]. However, enhanced cytotoxicity alone should not be taken as evidence of disulfidptosis without confirmation of the canonical biochemical and cytoskeletal features of the pathway.

Radiotherapy is also relevant because it can intensify NADPH demand and disulfide stress [229,243], potentially lowering the threshold for disulfidptotic injury. Because radiation generates ROS and increases the demand for thiol-dependent repair and antioxidant systems, tumors with high cystine uptake but limited NADPH regeneration may be particularly vulnerable to interventions that convert radiation-induced redox pressure into disulfide stress. Yet radiosensitization likewise requires careful distinction from other forms of radiation-induced damage.

Immunotherapy presents both promise and caution: tumor-cell disulfidptosis may improve antitumor responses, but metabolically stressed CD8^+^ T cells may also be vulnerable to related redox injury [112,241]. If disulfidptotic tumor-cell injury promotes immunogenic stress signals or reshapes the tumor microenvironment, it may theoretically enhance responsiveness to immune checkpoint blockade [241]. However, because activated T cells also require intact cysteine metabolism and redox buffering, therapeutic windows, dosing schedules, and tumor-selective delivery strategies will be critical [112].

Overall, the translational goal is not indiscriminate metabolic collapse, but the context-specific conversion of tumor redox liabilities into a selective therapeutic vulnerability. Future translational studies should therefore prioritize biomarker-guided patient selection, temporal scheduling of combination regimens, and parallel assessment of tumor-cell killing and immune-cell function [240,243].

### 9.2 Challenge of inducing disulfidptosis under physiologically realistic in vivo nutrient stress

A central translational challenge is that the most widely used in vitro trigger of disulfidptosis, near-complete glucose deprivation, does not faithfully reflect the nutrient conditions of most tumors in vivo [1,229]. Rather than uniform starvation, tumors experience heterogeneous states shaped by limited perfusion, hypoxia, stromal competition, and fluctuating nutrient supply. The key question is therefore not whether disulfidptosis can occur under extreme glucose-free conditions, but whether the same biochemical sequence can be triggered under physiologically plausible reductions in glucose and reducing power.

This distinction matters because tumor cells in vivo may compensate through glycogen mobilization, alternative NADPH-generating pathways, stromal support, or clonal adaptation [18,108,193]. Effective induction is therefore likely to require coordinated pressure on both cystine-disulfide loading and NADPH regeneration, rather than glucose restriction alone.

Future studies should define this metabolic context directly and should not infer disulfidptosis from tumor suppression alone. Essential validation includes evidence of disturbed cystine-cysteine and NADPH redox balance, disulfide accumulation, actin collapse, and pathway-specific rescue in physiologically relevant in vivo or ex vivo models. Only under these conditions can disulfidptosis be considered a credible therapeutic mechanism rather than an artifact of extreme in vitro starvation.

### 9.3 Translational priorities and future directions

Across all levels of evidence, the major translational challenge is not simply to induce tumor cell death under metabolically stressful conditions, but to demonstrate that the observed therapeutic effect truly derives from engagement of bona fide disulfidptosis rather than from broader metabolic collapse, oxidative injury, or overlap with other stress-associated death pathways [1,3]. Pathway engagement must also be evaluated as a dynamic process, as initially sensitive tumors may undergo metabolic and cytoskeletal adaptation under sustained therapeutic pressure. Advancing the field will therefore require a more rigorous framework for pathway attribution [3].

First, biomarker-guided patient selection should become a central principle in translational development. The strongest current rationale supports prioritization of SLC7A11-high tumors, particularly those with elevated cystine dependence and demonstrable vulnerability to glucose limitation or GLUT inhibition [1,18]. However, SLC7A11 expression alone is unlikely to be sufficient; future stratification models should also incorporate features of glucose transporter dependence, NADPH buffering capacity, redox adaptability, and cytoskeletal susceptibility, together with markers of adaptive rewiring, such as cystine handling, PPP activity, mitochondrial NADPH generation, redox buffering, and actin-network plasticity [1,3,18].

Second, adaptive resistance should be considered a central translational variable rather than a late-stage complication [229,244]. Most current models implicitly treat disulfidptotic susceptibility as a static metabolic state, whereas tumors are dynamic systems shaped by clonal selection, metabolic plasticity, and microenvironmental support [244]. Chronic disulfidptosis-inducing pressure may therefore select for resistant states through reduced cystine uptake, restored NADPH regeneration, enhanced redox buffering, or cytoskeletal remodeling, thereby shifting cells away from disulfidptosis toward other survival states or alternative death responses. Accordingly, acute sensitivity to glucose restriction or GLUT inhibition should not be assumed to predict durable therapeutic benefit [229].

Future translational studies should incorporate longitudinal resistance models rather than relying solely on acute treatment assays [241]. Approaches such as serial drug exposure, single-cell or spatial multi-omics, and redox-focused profiling may help define whether resistance arises from altered cystine loading, NADPH recovery, cytoskeletal adaptation, or stromal and immune support [240,243]. Such information will be important for designing rational combination strategies and for balancing tumor-directed disulfidptosis induction against potential injury to metabolically stressed CD8^+^ T cells in the tumor microenvironment [112,241,244].

Third, disease context matters. Most robust evidence currently comes from tumor models in which metabolic stress can be intentionally induced or therapeutically engineered [1]. Whether similar principles apply in non-cancer diseases remains an open question. As such, extrapolation across disease types should remain cautious unless supported by direct mechanistic validation [3]. In summary, disulfidptosis has emerged as a promising but still maturing translational concept. Current mechanistically supported studies provide initial preclinical proof-of-principle, particularly in SLC7A11-high, glucose-restricted tumor contexts and via engineered nanoplatforms that recapitulate the pathway's core biochemical triggers. Furthermore, broader exploratory data suggest a wider potential therapeutic landscape, though they underscore the ongoing need for strict mechanistic validation. Moving forward, the integration of biomarker selection, adaptive-resistance mapping, context-specific disease biology, and evidence-based pathway attribution will be necessary to determine whether disulfidptosis can successfully transition from an emerging concept to a clinically viable strategy.

## 10 Discussion

The conceptualization of disulfidptosis represents an important advance in understanding how metabolic rewiring can create selective cellular vulnerability. In malignancy, SLC7A11 overexpression supports cystine uptake and redox homeostasis, but it may also increase cellular susceptibility to disulfide stress under glucose-limited conditions. However, as the field continues to expand, the priority should shift from broadening the use of the term to defining its molecular boundaries and validation standards. Many reported disulfidptosis-related phenotypes are still based on indirect associations, such as SLC7A11 upregulation, NADPH depletion, intracellular cystine accumulation, cytoskeletal contraction, or prognostic gene signatures. These features are informative but not individually specific to disulfidptosis, because they may also accompany ferroptosis, apoptosis, necroptosis, or other forms of severe oxidative and metabolic stress. Therefore, future claims should be supported by integrated evidence, including cystine/SLC7A11 dependence, NADPH or GSH impairment, abnormal disulfide accumulation, actin cytoskeletal collapse, mechanism-relevant rescue, and exclusion of competing regulated cell death pathways.

A major unresolved issue is the causal relationship between disulfide stress and cytoskeletal failure. Current models link aberrant disulfide bond formation to collapse of the actin cytoskeleton, but the molecular sequence connecting these events remains incompletely defined. The key protein substrates that undergo lethal disulfide crosslinking have not been fully established, and it remains unclear whether F-actin collapse is the proximal execution mechanism or a prominent downstream manifestation of broader proteostatic failure. In addition, pathological disulfide formation may occur either broadly across the proteome or within specific subcellular microdomains, a distinction that has important mechanistic implications. Resolving these questions will require redox proteomics, site-specific cysteine reactivity profiling, live-cell spatiotemporal imaging, redox-resistant mutant rescue, and perturbation experiments that separate primary drivers from terminal correlates. Such approaches will be essential for distinguishing true disulfidptosis from disulfidptosis-like stress responses.

The physiological relevance of disulfidptosis also requires more rigorous validation in models that better reproduce in vivo metabolic and mechanical contexts. Most current evidence derives from highly sensitized cancer models exposed to severe glucose deprivation, whereas solid tumors and diseased tissues more commonly experience fluctuating hypoglycemia, hypoxia, matrix remodeling, and stromal interactions rather than complete glucose withdrawal. This distinction is critical because tissue-specific differences in glucose dependence, cystine flux, thiol-buffering capacity, NADPH regeneration, and cytoskeletal organization may determine the threshold for disulfidptosis induction. Traditional rigid two-dimensional culture systems may further distort this threshold, given that disulfidptosis is closely linked to actin architecture and cellular mechanics. Future studies should therefore incorporate patient-derived organoids, 3D biomimetic matrices, microfluidic nutrient-gradient systems, and immunocompetent in vivo models. These platforms will help determine whether disulfidptosis occurs under physiologically realistic low-glucose stress, whether it requires cooperating triggers such as hypoxia or extracellular matrix tension, and whether similar mechanisms operate in non-neoplastic diseases such as ischemia-reperfusion injury, neurodegeneration, or inflammatory tissue damage.

From a translational perspective, the major barrier is the lack of robust pharmacodynamic biomarkers and patient stratification strategies. SLC7A11 expression alone is unlikely to be sufficient for predicting disulfidptosis susceptibility, because pathway activation depends on the combined state of cystine uptake, glucose utilization, NADPH-regenerating capacity, thiol-buffering reserves, and cytoskeletal organization. Future biomarker panels should integrate these metabolic and structural parameters rather than relying on single-gene markers. In parallel, in vivo probes or biochemical assays capable of detecting aberrant disulfide-linked protein species will be needed to confirm target engagement and distinguish disulfidptosis from overlapping death programs. Moving forward, successful clinical translation will require a concerted shift from predominantly associative observations toward rigorous mechanistic validation in physiologically faithful models. Such evidentiary discipline will be essential to determine whether disulfidptosis can progress from an emerging cell death concept into a viable target for precision metabolic intervention.

## Ethics statement

Not applicable. (Review article).

## Funding

This work was supported by the Postgraduate Research & Practice Innovation Program of 10.13039/501100002949Jiangsu Province (KYCX25_4091, KYCX25_4095), the High-level Scientific and Technological Innovation Team Project of Kunshan Traditional Chinese Medicine Hospital (032025KCTD01), and the Kunshan High-level 10.13039/100018696Health Talent Plan (2025-4).

## CRediT authorship contribution statement

**Rongqing Li:** Conceptualization, Visualization, Writing – original draft. **Jiahui Wang:** Writing – review & editing. **Wei Li:** Funding acquisition, Supervision, Writing – review & editing. **Li Qian:** Funding acquisition, Project administration, Supervision, Writing – review & editing.

## Declaration of competing interest

The authors declare that they have no known competing financial interests or personal relationships that could have appeared to influence the work reported in this paper.

## Data Availability

No data was used for the research described in the article.
